# Transcriptome Profiling of *Chironomus kiinensis* under Phenol Stress Using Solexa Sequencing Technology

**DOI:** 10.1371/journal.pone.0058914

**Published:** 2013-03-20

**Authors:** Chuanwang Cao, Zhiying Wang, Changying Niu, Nicolas Desneux, Xiwu Gao

**Affiliations:** 1 Department of Forest Protection, Northeast Forestry University, Harbin, China; 2 Hubei Key Laboratory of Utilization of Insect Resources and Sustainable Control of Pests, Huazhong Agricultural University, Wuhan, China; 3 French National Institute for Agricultural Research (INRA), Sophia-Antipolis, France; 4 Department of Entomology, China Agricultural University, Beijing, China; Idaho State University, United States of America

## Abstract

Phenol is a major pollutant in aquatic ecosystems due to its chemical stability, water solubility and environmental mobility. To date, little is known about the molecular modifications of invertebrates under phenol stress. In the present study, we used Solexa sequencing technology to investigate the transcriptome and differentially expressed genes (DEGs) of midges (*Chironomus kiinensis*) in response to phenol stress. A total of 51,518,972 and 51,150,832 clean reads in the phenol-treated and control libraries, respectively, were obtained and assembled into 51,014 non-redundant (Nr) consensus sequences. A total of 6,032 unigenes were classified by Gene Ontology (GO), and 18,366 unigenes were categorized into 238 Kyoto Encyclopedia of Genes and Genomes (KEGG) categories. These genes included representatives from almost all functional categories. A total of 10,724 differentially expressed genes (*P* value <0.05) were detected in a comparative analysis of the expression profiles between phenol-treated and control *C. kiinensis* including 8,390 upregulated and 2,334 downregulated genes. The expression levels of 20 differentially expressed genes were confirmed by real-time RT-PCR, and the trends in gene expression that were observed matched the Solexa expression profiles, although the magnitude of the variations was different. Through pathway enrichment analysis, significantly enriched pathways were identified for the DEGs, including metabolic pathways, aryl hydrocarbon receptor (AhR), pancreatic secretion and neuroactive ligand-receptor interaction pathways, which may be associated with the phenol responses of *C. kiinensis*. Using Solexa sequencing technology, we identified several groups of key candidate genes as well as important biological pathways involved in the molecular modifications of chironomids under phenol stress.

## Introduction

Phenol, which comprises a benzene ring connected to a hydroxyl group, is commonly used in the production of dyes, polymers, drugs and other organic substances, such as pesticides, plastics and explosives [1], [2]. Phenol and its derivatives are widespread in aquatic ecosystems due to the runoff of water (containing pesticides and/or fertilizer residues) from agricultural fields into streams, as well as runoff from industrial and city waste sewage [3], [4]. As phenol and its derivatives are stable over the long term and are usually environmentally mobile, these compounds are considered to be major pollutants and acute and chronic toxicants. For example, phenol and its derivatives produce neurotoxic effects and cause liver and kidney damage and respiratory disorders [5]–[7]. The critical toxic concentration of phenol established by regulatory agencies varies widely worldwide, e.g., 105.24 µM in Malaysia [8], 10.63 µM in the United States [9] and 1.06 µM in Australia [10].

Phenol contamination of water ecosystems has serious environmental consequences due to the damaging effects of this compound on aquatic organisms [11], [12] such as algae and aquatic animals [13]. The importance of detecting the presence of phenol derivatives and assessing their impact on biota is acknowledged worldwide. As a result, a range of methodologies has been adopted. Direct chemical analysis is important due to the accuracy of this method, but direct chemical analysis has drawbacks, e.g., the requirement for complex sample pretreatment, expensive chemicals and expensive equipment [14]. In addition, this approach does not reveal temporal changes in exposure and/or the possible interactive effects of pollutants, nor does it provide ecologically relevant information [15]. To compensate for these limitations, various biological assays have been developed, including assays using aquatic animals, to provide information about pollutant-induced toxic effects and thereby assess environmental risks [16].

Midges, which belong to the chironomidae family, are among the most abundantly distributed groups of insects that can be used as bioindicators in freshwater ecosystems. Midges have a relatively short life cycle, which is mainly spent at the aquatic larval stage. In addition, midges are relatively sensitive to aquatic contaminants. Therefore, midges have been used extensively for acute, chronic and life-cycle bioassays in freshwater systems. Previous studies have demonstrated the effects of heavy metals [17] and pesticides [18]–[21] on the physiological, biochemical and molecular status of midges. Recently, we studied the impact of some substituted benzenes on chironomid populations at the ecological and biochemical levels [22], [23]. However, the cellular and molecular responses of chironomids to organic pollutants, especially for substituted benzenes, have rarely been studied.

Recently enhanced DNA sequencing platforms, such as the high-throughput Solexa/Illumina Genome Analyzer, provide a powerful method for assessing the relative importance of gene products in a given cell, tissue or organism [24], [25]. Cellular identity and function are determined by analyzing the transcriptome, i.e., the complete repertoire of RNA transcripts. The Solexa technique for transcriptome analysis can provide new information about whole-genome-wide transcript expression without prior sequence knowledge. This technique has been used to investigate human and mammalian diseases and functional genomics in plants and insects [26], [27]. Solexa transcriptome analysis of insects is a reliable and precise way to study genomic characteristics during development [28]–[31].

This is the first study in which transcriptome profiling analysis of *Chironomus kiinensis* was performed using RNA-sequencing (RNA-seq), and the identification of differentially expressed genes (DEGs) was used to gain a deeper understanding of the molecular toxicity of phenol. We used transcriptional profiling to identify key groups of genes and pathways that are differentially regulated in *C. kiinensis* under phenol stress. This study was performed to determine the molecular modifications of insects in response to phenol contamination in aquatic ecosystems.

## Materials and Methods

### Experimental midge rearing and sample preparation

The aquatic midge *Chironomus kiinensis* was obtained from Shenzhen Municipal Water Affairs Bureau and cultured using standard protocols with slight modifications [32]. Briefly, instead of collecting and separating egg masses, the midges were reared in mixed-age cultures in a glass chamber containing dechlorinated tap water and acid-washed sand with aeration (20±2°C and L16:D8) and fed goldfish granule food (Beijing SanYou Beautification Free TECH. CO., LTD, China). Healthy fourth-instar larvae of a similar size and color were used for the phenol stress tests. To obtain chironomid gene expression profiles, phenol stress and control cDNA samples were prepared from fourth larval *C. kiinensis* and sequenced using the Solexa platform. The larvae were collected with a pipette (visualized under a microscope) and transferred to a 1 L beaker containing 500 mL 10 µmol/L of phenol solution (or 500 mL dechlorinated tap water for the control). One hundred larvae were employed in each of three phenol treatment replicates and in the control over a period of 24 h. Fifteen surviving midges were randomly selected from each replicate for RNA preparation.

### RNA isolation and Solexa sequencing

Total RNA was isolated using an RNeasy Mini Kit (Qiagen) following the manufacturer's guidelines and treated with RNase free DNase I (Qiagen). RNA concentrations were measured using a spectrophotometer, and the RNA integrity was checked by analysis on a 1.0% (w/v) agarose gel. In brief, mRNA was purified from total RNA (a mixture of RNA from three replicates at equal ratios) using poly-T oligo magnetic beads and broken into small pieces using divalent cations at 94°C for 5 min. The first strand cDNA was synthesized using random primers, with cleaved mRNA fragments serving as templates. This process was followed by second-strand cDNA synthesis using DNA polymerase I and RNaseH. Illumina Solexa sequencing using the GAII platform was performed at the Beijing Genomics Institute (BGI; Shenzhen, China).

### Sequence assembly

Reads were assembled using Trinity [33]. The longest assembled sequences were referred to as contigs. The reads were then mapped back to contigs with paired-end reads to detect contigs from the same transcript and the distances between these contigs. Finally, sequences were obtained that lacked Ns and could not be extended on either end. Such sequences were defined as unigenes. The predicted amino acid sequences encoded by unigenes were aligned with sequences in protein databases such as NCBI Nr database, the Swiss-Prot Protein database, the KEGG pathway database and the Cluster of Orthologous Groups (COG) database using BLASTx (E-value<0.00001). Sequence orientations were determined according to the best match in the database. If the results from different databases conflicted with each other, a priority order of Nr, Swiss-Prot, KEGG and COG was followed when deciding the sequence direction of the unigenes. Orientation and protein coding region prediction (CDS) of sequences having no hits in BLAST were predicted using ESTScan [34]. Original transcript sequences (5′->3′) were provided if their orientations could be determined. Other sequences were provided by the assembler outputs.

### Sequence annotation

Unigene annotations provide functional annotations for all unigenes, along with their expression levels. Functional annotations of unigenes were analyzed using protein sequence similarity, KEGG Pathway, COG and GO analysis. All unigene sequences were against the protein databases (Nr, SwissProt, KEGG, COG) using BLASTx (E-value<0.0001). Protein function information could be predicted from annotations of the most similar proteins in the databases. The KEGG pathway database records networks of molecular interactions in the cells, and variants of these pathways are specific to particular organisms. COG is a database where orthologous gene products are classified. The assumption is that every protein has evolved from an ancestor protein, and the entire database is built on coding proteins with complete genomes as well as system evolutionary relationships between bacteria, algae and eukaryotes. All unigenes were aligned to the COG database to predict and classify their possible functions. GO functional annotation was obtained from Nr annotation. GO annotation comprises three ontologies, i.e., a molecular function, a cellular component and a biological process. The basic GO unit is a GO-term, and every GO-term belongs to a type of ontology. Using Nr annotation, the Blast2GO program was used to obtain GO annotations of all of the unigenes [35], [36]. Blast2GO has been cited more than 150 times in other reports and is a widely recognized GO annotation program. After obtaining GO annotation for every unigene, Web Gene Ontology Annotation Plot (WEGO) software was used to carry out GO functional classification for all unigenes and to characterize the distribution of gene functions in the species gene functions at the macro level [37].

### Digital gene expression library preparation and analysis

Gene expression levels were calculated using the Reads Per kb per Million reads (RPKM) method [38]. Unigene expression levels were calculated according to the formula RPKM = (1,000,000*C) / (N*L*1,000), where RPKM (A) represents the expression of gene A, C is the number of reads that uniquely aligned to gene A, N is the total number of reads that uniquely aligned to all genes and L is the number of bases in gene A. The RPKM method succeeded in eliminating the influences of different gene lengths and sequencing discrepancy on the calculation of gene expression level. Therefore, the calculated gene expression level could be used to directly compare the level of gene expression among samples. If there was more than one transcript for a given gene, the longest transcript was used to calculate its expression level and coverage. To identify DEGs between phenol-treated and control samples, the false discovery rate (FDR) method was used to determine the threshold of P-value in multiple tests [39]. The significance of difference in gene expression was judged using a threshold FDR≤0.001 and an absolute value of log_2_Ratio≥1. Then, the genes expressed at different levels across samples were further annotated by GO enrichment analysis and KEGG pathway enrichment analysis.

### Real-time RT-PCR analysis

Approximately 1 µg of total RNA was reverse transcribed to cDNA using 1 µM of oligodeoxythymidine primer. The synthesized cDNAs were diluted to 100 µL with sterile water and used as the template for real-time PCR. Twenty genes that showed expression differences (ten upregulated genes and ten downregulated genes) between the two libraries were randomly selected for validation. The primer sequences are listed in Table 1. Real-time RT-PCR was performed in an MJ Opticon™^2^ machine (Bio-Rad, Hercules, CA, USA). The actin and TAU genes were chosen as internal controls to normalize the amount of total RNA present in each reaction. The reaction mixture (20 µL) contained 10 µL of SYBR Green Realtime PCR Master Mix (Toyobo), 0.5 µM each of forward and reverse primers and 2 µL of cDNA template (equivalent to 100 ng of total RNA). The amplifications were performed with the following parameters: 94°C for 30 s followed by 45 cycles at 94°C for 12 s, 60°C for 30 s, 72°C for 40 s and 82°C for 1 s for plate reading. A melting curve was generated for each sample at the end of each run to assess the purity of the amplified products. Real-time PCR was carried out in triplicate (technical repeats) to ensure the reproducibility of the results. The expression levels of the clones were calculated from the threshold cycle according to the delta-delta CT method [40]. The relative expression level was calculated by dividing the transcription level under phenol stress conditions by the transcription level under control conditions. All of the relative expression levels were log2 transformed.

**Table 1:** Primers used in Real-time RT-PCR.

Gene number	Forward primer (5′-3′)	Reverse primer (5′-3′)
U18403	TTGTGAACGAGCAGGAACGAGT	GTCGTTATCCATGCGTGTTGTG
U9085	AACCATCGTTTCATGCCGTCCA	GCATCATCTTCATCAACGCAGC
U19940	CAATTTATCAACGCCGGTCCGA	TCATCACAATCTGGATTCATGTCAGT
U20077	AGACACAACAACATCCTCGCCA	TCCTGTGTGTGTGTGTGTGTTC
U19007	CATTCTCTTCGCAACAATGATGCTT	AAGCTTTGTTTGCTCGGACTCACC
U14484	GGACCTGCAAAGGAGGAGAATA	GCTGGAGGCACTGCGATTCAATTA
U17324	ATCGTTTGCCGCACGAAATCCT	AATTGTGTCCTGTACGCCCGTT
U18859	TAGTTTGCTTGTCATGTCCGGTCC	CCCGAAGGATCGGAATGTTTGT
U10424	AGCGATGGCCGATATGGAAGAA	ACTGTTGTAAGCACTCGAACCC
U13743	TGTCGCATCGGACAACAATCCA	CAAGCATCTTCAGCGGGTCTTT
U6658	TGCTCGTTCTTGCTGACTGTGT	TTGGTGAATGGCCCAGTTGGAT
U449	GCTCAAGCATGATCTGGAAACC	TGTGTATGCTCTTGTGCTCCCA
U3979	TTCACCACCAGCACAGAGTTGA	GCCAGTCTGCTTGCCATTAAGT
U6040	ATTTGGCATTGGAGTTGGGACC	ACTCATCAACCCACCCTCAACA
U3434	TCGCCCACATGTCTTTGACCAT	TGTTTGTCCCATGGGCTGATGT
U1928	TCCATCTTGTGCTCCAACTGCT	GACCGCTTAGACTTCATAGAGT
U7314	CGCTGTGGTCATTGACGAGAAT	GCTAGAGATCGATCATTGCCTG
U7204	ATTGCTGGCTTCACCATCACCT	CGTCAGCAACGCAGCAAAGTTG
U6065	ATGCTGTTGAGGGTGGCAATGA	ACCTCTTGCGTTGACCCATCTA
U1488	AGGGTGCCTCGACCTTCTTCA	TCTGGATGTTGGTGGTGTCCAA
Actin	AATGGGATCGCTTGGGTGCTTT	TCAGCTTCACCCAATGTTGCCT
TAU	TGGTGGCGACAAGCGCATTATT	TGGCGTTATAGGAACCGGATGT

## Results

### Tag identification and quantification

After cleaning and quality checks, we obtained 51,518,972 reads with a mean length of 84 bp for the phenol treatment library (PT) and 51,150,832 million reads with a mean length of 85 bp for the control library (CK). These raw reads were further assembled into contigs using Trinity software, resulting in 98,194 contigs with a mean length of 338 bp and 79,786 contigs with a mean length of 360 bp in the PT and CK libraries, respectively (Table 2). The size distribution of these contigs is shown in Fig. S1. In this study, we obtained 55,338 unigenes with a mean length of 589 bp for the PT library and 49,804 unigenes with a mean length of 568 bp for the CK library.

**Table 2:** Summary for the *Chironomus kiiensis* transcriptome in phenol treated (PT) and control (CK) libraries.

	PT	CK
Total number of reads	54,894,824	54,038,722
Total base pairs (bp)	4,636,707,480	4,603,574,880
Average read length(bp)	84	85
Total number of contigs	98,194	79,786
Mean length of contigs(bp)	338	360
Distinct clusters	9,777	7,623
Distinct singletons	45,561	42,181
Total distinct sequences	55,338	49,804
Mean length of unigene (bp)	589	568
Sequences with E-value<10^−5^	25,239	

### Annotation of predicted proteins

For annotation, distinct gene sequences were first searched using BLASTx against the non-redundant (Nr) NCBI nucleotide database, with a cut-off E-value of 10^−5^. Using this approach, 25,239 genes (87.39% of all distinct sequences) returned an above cut-off BLAST result (Table S1). Table 3 indicates that the proportion of shorter assembled sequences with matches in the Nr database was greater than that in the other databases. A total of 59.47% of the matches were observed for sequences ranging from 100 to 500 bp, whereas the match efficiency decreased to 21.00% for those ranging from 500 to 1,000 bp and 6.51% for sequences longer than 2,000 bp (Table 3).

**Table 3:** Length distribution of non-redundant consensus sequences.

Length of non-redundant unigenes	Total Number	Percentage
100–500	30,336	59.47%
500–1000	10,714	21.00%
1000–1500	4,350	8.53%
1500–2000	2,294	4.50%
> = 2000	3,320	6.50%
All unigenes	51,014	
Length of all unigenes (nt)	37044122	
N50 (bp) *	1137	
Mean Size (bp)**	726	

* N50 = median length of all unigenes; **Mean size = average length of all unigenesGO and COG classification

**Figure 1:**
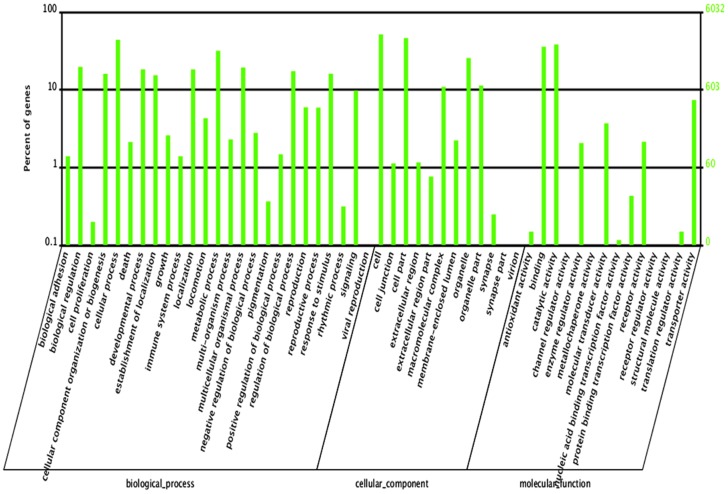
GO annotations of non-redundant consensus sequences. Best hits were aligned to the GO database, and 6032 transcripts were assigned to at least one GO term. Most consensus sequences were grouped into three major functional categories, namely biological process, cellular component, and molecular function.

**Figure 2:**
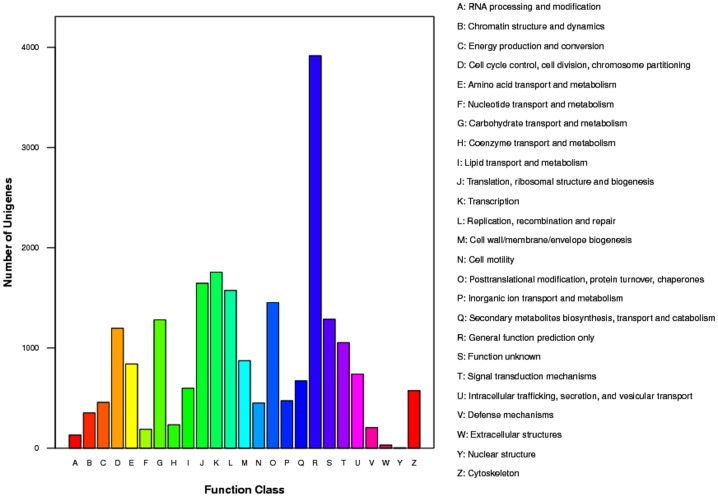
COG annotations of putative proteins. All putative proteins were aligned to the COG database and can be classified functionally into at least 25 molecular families.

GO assignments were used to classify the functions of the predicted *C. kiinensis* genes. Based on sequence homology, 6,032 sequences were categorized into 51 functional groups (Fig. 1). To further evaluate the completeness of the transcriptome library and the effectiveness of the annotation process, we searched the annotated sequences for the genes involved in COG classifications. In total, 9,758 sequences had COG classifications (Fig. 2). Among the 25 COG categories, the cluster for “General function prediction” represented the largest group (17.82%), followed by “Transcription” (7.99%) and “Translation, ribosomal structure and biogenesis” (7.49%; Fig. 2). These functional annotations provide valuable information that helps elucidate the biological processes, functions and pathways employed by *C. kiinensis* in response to phenol stress.

### Comparison of DEG level between the two libraries

**Figure 3:**
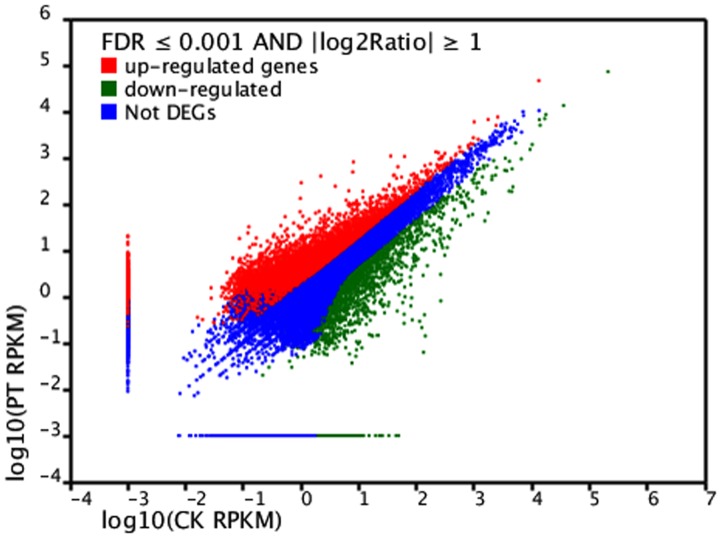
Comparison of gene expression level between the two libraries. For comparing gene expression level between the two libraries, each library was normalized to 1 million tags. Red dots represent transcripts more prevalent in the phenol treatment library, green dots show those present at a lower frequency in the infected tissue and blue dots indicate transcripts that did not change significantly. The parameters “FDR<0.001” and “log2 Ratio≥1” were used as the threshold to judge the significance of gene expression difference.

**Figure 4:**
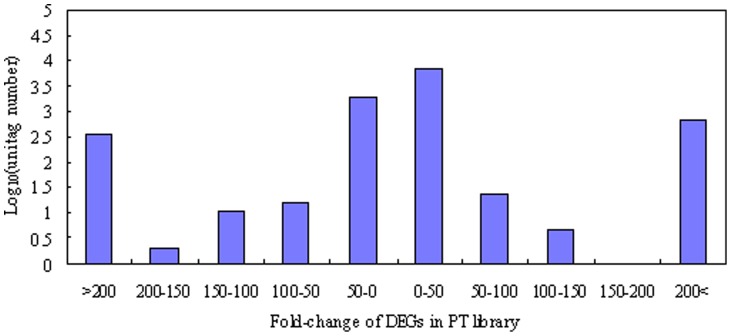
Differentially expressed genes in phenol tissue library. The “X” axis represents fold-change of DEGs in the PT library. The “y” axis represents the number of unique tags (log 10).

Differences in the tag frequencies appearing in the PT and CK libraries were used to estimate the gene expression levels in response to phenol stress. The transcripts detected with at least two-fold differences (FDR<0.001 AND |log_2_^Ratio^|≥1) in the two libraries are shown in Fig. 3. The red dots (8,390) and green dots (2,334) represent transcripts with higher or lower abundance (more than two-fold), respectively, in the CK library. The blue dots represent transcripts that differed less than two-fold between the two libraries, which were arbitrarily designated as “no difference in expression”. The DEGs with five-fold or greater differences in accumulation are shown in Fig. 4. A total of 3,121 genes exhibited increased expression of at least five-fold in the PT library, while 1,139 genes exhibited at least a five-fold decrease in expression in the PT library compared with the CK library. The expression levels of 60.28% of the unique tags were within a five-fold difference in range between the PT and CK libraries. DEGs with differences greater than 20-fold are showed in Table S2. Finally, 176 upregulated and 294 downregulated genes in the PT library exhibited a 20-fold difference in expression compared with those in the CK library.

### Real-time RT-PCR analysis

To validate the Solexa expression profiles, 20 genes were randomly selected for transcript levels analysis. Among these, ten genes (U18403, U9085, U19940, U20077, U19007, U14484, U17324, U18859, U10424 and U13743) were upregulated, and ten genes (U6658, U449, U3979, U6040, U3434, U1928, U7314, U7204, U6065 and U1488) were downregulated (Table 4). Actin and TAU, reported to be stably expressed in insects, were chosen as reference genes for data normalization. The trend of RT-PCR based expression profiles among these selected genes was similar to those detected by the Solexa-sequencing based method. However, the scale of the differences in expression between genes in the PT and CK libraries detected by real-time PCR was generally smaller than that detected by the Solexa sequencing-based method (Table 4).

**Table 4:** Genes selected for Real-time RT-PCR.

Gene number	Description	RT-PCR fold	Solexa fold
U18403	Cytochrome b561 domain-containing protein 1 [*Harpegnathos saltator*]	8.8	13.9
U9085	glutathione s-transferase [*Chironomus riparius*]	10.6	13.0
U19940	melanization protease 1, isoform C [*Drosophila melanogaster*]	11.9	12.3
U20077	T-cell receptor beta chain ANA 11 [*Brugia malayi*]	3.1	11.8
U19007	sodium/solute symporter [*Culex quinquefasciatus*]	8.8	11.4
U14484	sodium/shloride dependent amino acid transporter [*Aedes aegypti*]	6.5	11.0
U17324	environmental stress-induced protein [*Culex quinquefasciatus*]	1.7	9.9
U18859	cytochrome P450 349A1 [*Tribolium castaneum*]	9.3	8.9
U10424	transmembrane and coiled-coil domains protein 1 [*Culex quinquefasciatus*]	3.1	4.8
U13743	N-acetyl lactosaminide beta-1,3-N-acetyl glucosaminyl transferase [*Culex quinquefasciatus*]	3.1	4.5
U6658	alkaline phosphatase [*Culex quinquefasciatus*]	−3. 2	−5.1
U449	nicotinic acetylcholine receptor, beta-2 subunit [*Culex quinquefasciatus*]	−2.4	−5.3
U3979	Serine protease easter [*Harpegnathos saltator*]	−4.9	−9.5
U6040	lung carbonyl reductase [*Aedes aegypti*]	−6.0	−10.0
U3434	S-phase kinase-associated protein 1 [*Bubalus bubalis*]	−8.2	−10.3
U1928	leucine-rich transmembrane protein [*Aedes aegypti*]	−9.1	−10.6
U7314	N-acetyl galactosaminyl transferase 6 [*Culex quinquefasciatus*]	−7.1	−11.7
U7204	voltage gated chloride channel domain-containing protein [*Toxoplasma gondii* ME49]	−2.3	−12.1
U6065	CG3884, isoform C [*Drosophila melanogaster*]	−8.4	−13.9
U1488	Wiskott-Aldrich syndrome, like [*Danio rerio*]	−6.4	−15.6

### Pathways enrichment analysis of DEGs

To identify the biological pathways in *C. kiinensis*, we mapped the 8,146 annotated sequences to the reference canonical pathways in KEGG. In total, we assigned 18,366 sequences to 238 KEGG pathways. Significantly enriched metabolic pathways and signal transduction pathways were identified. A total of 238 pathways were affected by 5,091 upregulated DEGs and 3,055 downregulated DEGs (Table S3). Pathways with Q value <0.05 were significantly enriched. DEG enrichment analysis showed that the first three pathways involving upregulated genes (in response to phenol stress) were metabolic pathway (336), regulation of actin cytoskeleton (106) and focal adhesion (91). By contrast, the first three pathways involving in downregulated genes were metabolic pathways (283), pancreatic secretion (122) and protein digestion and absorption (101; Table S3). These annotations provide a valuable resource for investigating specific processes, functions and pathways for further research of chironomids under phenol stress.

### AhR-mediated defense genes associated with phenol stress

The aryl hydrocarbon receptor (AhR) is a soluble, ligand-dependent transcription factor that belongs to the basic helix-loop-helix-PAS family of regulatory proteins. AhR regulates the expression of defense genes in invertebrates in response to organic pollutants. However, in invertebrates, proteins encoded by genes with functions similar to that of AhR, including spineless (*Ss*) and single-minded (*Sim*), can heterodimerize with the aryl hydrocarbon receptor nuclear translocator (ARNT) ortholog tango (*Tgo*) to regulate the expression of downstream genes. Interestingly, DEG analysis revealed a battery of *AhR* genes that showed different levels of induction or inhibition under phenol stress. *AhR* homolog spineless (*Ss*) and ARNT ortholog tango (*Tgo*) in PT library showed 1.85- and 2.53-fold increased expression in the PT library vs. the CK library, respectively. Moreover, three *AhR* ortholog single-minded genes were also induced, but not significantly, in the PT library compared with the CK library. Some AhR-mediated downstream genes were significantly induced or inhibited in the PT library. For example, 28 P450 genes were downregulated in the PT library (ranging from 4-fold to 5,000-fold downregulation), while sixteen of these genes were upregulated in the PT library (ranging from 12-fold to 1229-fold upregulation). Twenty-two genes encoding heat shock proteins (HSPs) showed a four-fold difference in expression in the PT library vs. the CK library, including 8 downregulated HSPs genes and 14 up-regulated HSPs genes. Among 26 genes encoding UDP glucuronosyltransferase, six were downregulated in the PT library (ranging from 4.9 to 1,000-fold), while 20 genes were upregulated (ranging from 3.5 to 239.9-fold; Table 5).

**Table 5:** AhR-mediated defense genes associated with phenol stress.

Gene	Gene ID	Gene name	Fold (PT/CK)
Aryl hydrocarbon receptor (AhR)	CL682.Contig1	Ahr homolog spineless [*Drosophila melanogaster*]	1.851
	Unigene5384	tango [*Drosophila melanogaster*]	2.533
Cytochrome P450	Unigene1523	cytochrome P450 6BK11 [*Tribolium castaneum*]	0.000205
	Unigene7608	Cyp313a1 [*Drosophila melanogaster*]	0.000450
	Unigene4583	cytochrome p450-like protein [*Leishmania braziliensis* MHOM/BR/75/M2904]	0.00148
	Unigene4751	cytochrome P450-28A1 [*Drosophila mettleri*]	0.00156
	Unigene2527	PREDICTED: cytochrome P450 2B19-like [*Xenopus (Silurana) tropicalis*]	0.00157
	Unigene4608	cytochrome P450 reductase C [*Trypanosoma cruzi*]	0.00190
	Unigene2199	cytochrome P450 [*Aedes aegypti*]	0.00318
	Unigene414	cytochrome P450 93A3 [*Culex quinquefasciatus*]	0.011
	Unigene5277	cytochrome P450 [*Culex pipiens pallens*]	0.015
	CL2881.Contig1	cytochrome P450 [*Aedes aegypti*]	0.065
	CL3766.Contig1	cytochrome P450 4d8 [*Culex quinquefasciatus*]	0.066
	Unigene1681	cytochrome P450 [*Aedes aegypti*]	0.073
	Unigene4988	cytochrome P450 [*Aedes aegypti*]	0.098
	Unigene1854	cytochrome P450 [*Aedes aegypti*]	0.108
	CL8733.Contig1	cytochrome P450 [*Aedes aegypti*]	0.109
	CL14278.Contig1	corpora allata cytochrome P450 [*Diploptera punctata*]	0.114
	Unigene8409	cytochrome P450 [*Aedes aegypti*]	0.126
	Unigene4683	cytochrome P450 [*Aedes aegypti*]	0.129
	CL12023.Contig1	cytochrome P450 [*Aedes aegypti*]	0.135
	CL16912.Contig1	cytochrome P450 4d8 [*Culex quinquefasciatus*]	0.140
	CL1838.Contig1	cytochrome P450 [*Aedes aegypti*]	0.166
	Unigene16823	cytochrome P450 CYP4D4v2 [*Musca domestica*]	0.171
	Unigene2056	cytochrome P450 6d3 [*Culex quinquefasciatus*]	0.171
	CL15542.Contig1	cytochrome P450 [*Culex pipiens pallens*]	0.173
	Unigene13399	PREDICTED: cytochrome P450 307a1 [*Apis mellifera*]	0.176
	CL2051.Contig1	cytochrome P450 [*Aedes aegypti*]	0.194
	Unigene682	cytochrome P450 [*Aedes aegypti*]	0.229
	CL5447.Contig1	Cytochrome P450 6B3 [*Acromyrmex echinatior*]	0.248
	Unigene19789	cytochrome P450 26B1 [*Culex quinquefasciatus*]	11.654
	Unigene19547	cytochrome P450 CYP6Z2 [*Anopheles gambiae*]	12.335
	Unigene13715	cytochrome P450 6a22 [*Culex quinquefasciatus*]	22.615
	Unigene17385	cytochrome P450 [*Anopheles gambiae*]	24.161
	Unigene9177	cytochrome P450 CYP6Z2 [*Anopheles gambiae*]	28.790
	Unigene11217	cytochrome P450 CYP6Z2 [*Anopheles gambiae*]	41.124
	CL24894.Contig1	cytochrome P450 [*Aedes aegypti*]	51.357
	Unigene18455	cytochrome P450 [*Anopheles minimus*]	61.185
	CL16599.Contig1	cytochrome P450 [*Leptinotarsa decemlineata*]	71.546
	Unigene6768	cytochrome P450 [*Aedes aegypti*]	170.296
	Unigene10912	cytochrome P450 [*Aedes aegypti*]	177.196
	Unigene18859	cytochrome P450 349A1 [*Tribolium castaneum*]	463.010
	Unigene16330	cytochrome P450 6BK11 [*Tribolium castaneum*]	499.105
	Unigene12383	cytochrome P450 4d8 [*Culex quinquefasciatus*]	825.200
	Unigene9491	cytochrome P450 *[Aedes aegypti*]	913.018
	Unigene8827	cytochrome P450 6a22 [*Culex quinquefasciatus*]	1228.771
Heat shock protein (HSP)	Unigene3437	heat shock protein cognate 1, isoform A [*Drosophila melanogaster*]	0.00066
	Unigene4224	Hsp70 protein [*Mitsukurina owstoni*]	0.00088
	Unigene2422	stress protein HSP70-1 [*Xiphophorus maculatus*]	0.00104
	Unigene5685	HSP70-like protein [*Philodina roseola*]	0.00192
	Unigene812	HSP70 [*Bodo saltans*]	0.11422
	CL23847.Contig1	Hsp67Bb [*Drosophila yakuba*]	0.15966
	Unigene5109	heat shock protein HSP70 [*Trypanosoma cruzi*]	0.18696
	Unigene7592	Hsp26 [*Drosophila buzzatii*]	0.19571
	Unigene10483	Hsp70-interacting protein [*Glossina morsitans morsitans*]	4.112
	Unigene15875	heat shock protein hsp20.1 [*Bombyx mori*]	4.113
	CL24088.Contig1	HSP70 [*Chironomus tentans*]	4.317
	Unigene9955	PREDICTED: similar to DnaJ (Hsp40) homolog, subfamily C, member 11 [*Tribolium castaneum*]	4.627
	CL1076.Contig1	HSP70 [*Chironomus tentans*]	4.707
	CL4385.Contig1	HSP70 [*Chironomus tentans*]	5.220
	CL18832.Contig1	23kDa heat shock protein ScHSP23 [*Sarcophaga crassipalpis*]	5.240
	CL18990.Contig1	heat shock protein Hsp20 [*Liriomyza huidobrensis*]	5.412
	CL20479.Contig1	DNA-J/hsp40 [*Aedes aegypti*]	5.492
	Unigene12651	heat shock protein 27 [*Drosophila melanogaster*]	5.610
	CL18851.Contig1	Hsp26 [*Drosophila buzzatii*]	6.740
	CL22167.Contig1	Hsp26 [*Drosophila buzzatii*]	7.709
	CL20629.Contig1	HSP70 [*Chironomus tentans*]	8.411
	CL24931.Contig1	DnaJ (Hsp40) homolog, subfamily B, member 9 [*Schistosoma japonicum*]	14.510
Glutathione-S- transferase (GST)	Unigene7559	glutathione-s-transferase theta, gst [*Culex quinquefasciatus*]	0.10797
	Unigene20227	glutathione-s-transferase theta, gst [*Culex quinquefasciatus*]	0.12839
	Unigene5677	glutathione-s-transferase theta, gst [*Culex quinquefasciatus*]	0.17135
	CL19658.Contig1	glutathione transferase, microsomal (AGAP000165-PA) [*Anopheles gambiae* str. PEST]	3.20495
	Unigene3057	delta GST [*Mayetiola destructor*]	5.14118
	CL28765.Contig1	microsomal glutathione transferase GSTMIC2 [*Culex quinquefasciatus*]	5.29525
	Unigene18888	glutathione transferase, microsomal (AGAP000165-PA) [*Anopheles gambiae* str. PEST]	3.20495
	Unigene9090	RecName: Full = Glutathione S-transferase; AltName: Full = GST class-sigma	3.50157
UDP-glucuronosyltransferase	Unigene871	UDP-glucosyltransferase [*Bombyx mori*]	0.0010
	Unigene6169	glucosyl/glucuronosyl transferases [*Aedes aegypti*]	0.0366
	Unigene1960	PREDICTED: UDP-glucuronosyltransferase 2B13-like [*Acyrthosiphon pisum*]	0.0447
	Unigene7940	UDP-glucuronosyltransferase 2C1 [*Culex quinquefasciatus*]	0.1341
	Unigene2200	PREDICTED: UDP-glucuronosyltransferase 2A3-like [*Nasonia vitripennis*]	0.1446
	CL10485.Contig1	UDP-glucuronosyltransferase 2B28 [*Culex quinquefasciatus*]	0.2057
	CL9485.Contig1	UDP-glucuronosyltransferase 2C1 [*Culex quinquefasciatus*]	3.5296
	CL6867.Contig1	UDP-glucuronosyltransferase R-21 [*Culex quinquefasciatus*]	3.5546
	CL5011.Contig1	UDP-glucuronosyltransferase [*Culex quinquefasciatus*]	3.6823
	CL12598.Contig1	UDP-glucuronosyltransferase R-21 [*Culex quinquefasciatus*]	3.6961
	CL28510.Contig1	PREDICTED: UDP-glucuronosyltransferase 2B20-like isoform 2 [*Acyrthosiphon pisum*]	4.2945
	CL23086.Contig1	UDP-glucuronosyltransferase 2B20 [*Culex quinquefasciatus*]	4.3484
	Unigene14475	UDP-glucuronosyltransferase 2B20 [*Culex quinquefasciatus*]	4.6268
	CL22005.Contig1	UDP-glucuronosyltransferase 2B1 [*Culex quinquefasciatus*]	5.1152
	CL267.Contig1	UDP-glucuronosyltransferase 2B4 [*Culex quinquefasciatus*]	5.3987
	Unigene14342	UDP-glucuronosyltransferase 2B15 [*Culex quinquefasciatus*]	6.6830
	CL3387.Contig1	UDP-glucuronosyltransferase 2B15 [*Culex quinquefasciatus*]	8.1038
	Unigene10740	UDP-glucuronosyltransferase 2B20 [*Culex quinquefasciatus*]	9.9397
	Unigene12082	UDP-glucuronosyl transferase [*Aedes aegypti*]	16.6241
	Unigene11492	UDP-glucuronosyltransferase R-21 [*Culex quinquefasciatus*]	75.0666
	Unigene19976	UDP-glucuronosyltransferase 2B20 [*Culex quinquefasciatus*]	283.4008
	Unigene5157	Methylmalonate-semialdehyde dehydrogenase [*Salmo salar*]	0.00209
	Unigene3952	alcohol dehydrogenase class-3 [*Oryctolagus cuniculus*]	0.093
	CL27274.Contig1	aldehyde dehydrogenase [*Aedes aegypti*]	2.806
	Unigene19683	acyl-CoA synthetase family member 4 [*Mus musculus*]	4.884
	Unigene17726	formaldehyde dehydrogenase [*Drosophila melanogaster*]	239.902
NADH-Ubiquinone oxidoreductase	Unigene3047	NADH-ubiquinone oxidoreductase NDUFS3/30 kDa subunit [*Glossina morsitans morsitans*]	0.176
	Unigene4631	NADH-Ubiquinone oxidoreductase AGGG subunit [*Pediculus humanus corporis*]	0.206
	Unigene6983	NADH-Ubiquinone oxidoreductase AGGG subunit [*Pediculus humanus corporis*]	0.309
	Unigene21733	NADH-plastoquinone oxidoreductase [*Aedes aegypti*]	2.111
	CL4001.Contig1	RecName: Full = NADH-ubiquinone oxidoreductase chain 5; AltName: Full = NADH dehydrogenase subunit 5	2.654
	CL2952.Contig1	PREDICTED: quinone oxidoreductase-like [*Nasonia vitripennis*]	2.970
	CL15165.Contig1	NADH-ubiquinone oxidoreductase chain 2 [*Simulium nigrimanum*]	3.085

## Discussion

Phenol, also known as carbolic acid or phenic acid, is a strong neurotoxin. Exposure to phenol can cause instant death, as it shuts down the neural transmission systems [14]. Phenol can induce harmful effects on the central nervous system and heart, causing dysrhythmia, seizures and ultimately, a coma [41]. Moreover, exposure to phenol through skin contact or by inhalation and is toxic, as phenol is corrosive to the eyes, skin and respiratory tract, and exposure can lead to lung edema [42]. In this study, we identified multiple DEGs and signal pathways involved in the responses to short-term exposure to phenol in an aquatic arthropod model, *C. kiinensis*.

### AhR pathway associated with phenol stress

AhR is a member of the family of basic helix-loop-helix (bHLH) transcription factors [43]. The physiological ligands of AhR are unknown, but this transcription factor can bind to several exogenous ligands such as plant flavonoids and synthetic polycyclic aromatic hydrocarbons. AhR is a cytosolic transcription factor that is normally inactive (i.e., bound to several molecular chaperones). When the ligand is bound to chemicals such as 2,3,7,8-tetrachlorodibenzo-p-dioxin (TCDD), the chaperones dissociate, and AhR translocates into the nucleus and dimerizes with ARNT. This leads to changes in gene transcription. Both mammalian and arthropod proteins belong to the bHLH-PAS class of proteins, which have a bHLH DNA binding domain, a Per-ARNT-Sim (PAS) protein-protein interaction and ligand-binding domains [44]–[46]. In the developmental pathway of insects, the AhR orthologs, spineless (*Ss*) and single-minded (*Sim*), heterodimerize with the ARNT ortholog tango (*Tgo*) in the cytosol without binding to a ligand. These heterodimers translocate to the nucleus where Ss/Tgo preferentially binds to XRE–AhR, and Sim/Tgo binds to the central midline element (CME). To date, six downstream genes regulated by AhR have been found in vertebrates, including genes encoding CYP1A1, CYP1A2, NAD(P)H: quinone oxidoreductase, aldehyde dehydrogenase 3, UDP glucuronosyltransferase and glutathione transferase [47]–[50]. In this study, we identified a set of genes in *C. kiinensis*, i.e., genes encoding AhR orthologs (*Ss* and *Sim*) and ARNT ortholog (*Tgo*), that are similar to mammalian AhR pathway genes. Similarly, a battery of AhR genes was found to be either upregulated or downregulated in *C. kiiensis* under phenol stress. These results indicate that the AhR pathway in chironomids may be involved in the response to phenol stress and thus may be an important biological pathway. However, further studies are needed to investigate the regulatory role of the AhR pathway in insects facing phenol stress.

### Pancreas pathway and phenol stress

The pancreas performs both exocrine and endocrine functions. The exocrine pancreas is composed of two functional parts, the acinar and duct cells. The primary functions of pancreatic acinar cells are to synthesize and secrete digestive enzymes. Stimulation of the cells by secretagogs such as acetylcholine (ACh) and cholecystokinin (CCK) creates an intracellular Ca^2+^ signal. This signal, in turn, triggers the fusion of the zymogen granules with the apical plasma membrane, leading to polarized enzyme secretion. The major task of pancreatic duct cells is the secretion of fluids and bicarbonate ions (HCO_3_^−^), which neutralize the acidity of gastric contents entering the duodenum. An increase in intracellular cAMP is one of the major signals of HCO_3_^−^ pancreatic secretion. Activation of the CFTR Cl^−^ channel and CFTR-dependent Cl^−^/HCO_3_^−^ exchange activities is responsible for cAMP-induced HCO_3_^−^ secretion. Thus, pancreatic secretion is another important pathway in the biological process category. In this study, we found that 87 upregulated genes and 122 downregulated genes by phenol stress are involved in the pancreatic secretion process (Table 6). Among these DEGs, Ras-related C3 botulinum toxin substrate 2 precursor (Rac2) and RAS-like protein were significantly inhibited by phenol compared with the control. Rac 2 protein belongs to the GTP-binding proteins of the Rho family; there is an alternating regulatory cycle between the active GTP-bound form and the inactive GDP-bound form of Rac 2. This cycle involves three distinct families of proteins, i.e., guanine exchange factors (GEFs), GTPase-activating proteins (GAPs) and guanine nucleotide dissociation inhibitors (GDIs). Upon loading GTP, a conformational change takes place that allows Rac2 protein to interact with several downstream effectors. Ultimately, this information is processed, and the signal is propagated the signal within the cell, causing changes to the actin cytoskeleton, the release of inflammatory modulators and innate immunity. The signaling of active Rac2 is mediated through its interaction with effectors such as p67phox and cytochrome b-558 [51], PLCbeta2 [52], nitric oxide synthase 2 (NOS_2_) [53] and Pak1 [54]. Expression is restricted to hematopoietic cells, with the highest levels of expression in myeloid cells. Rac2 expression is regulated during the differentiation of hematopoietic and myeloid cells, and some data suggest that Rac2 might be expressed in tumors. As suggested by its restricted expression, Rac2 plays a specialized role in many hematopoietic and immunological processes. Rac2-deficient mice show defects in stem cells, mast cells and B and T cells.

**Table 6:** Genes in pancreatic secretion pathway.

Description	Gene symbol	Ratio
Ras-related C3 botulinum toxin substrate 2 precursor	*Rac2*	−3323.07
RAS-like protein	*Rap*	−1437.25
Pancreatic lipase-related protein 1-like	*PLRP1*	−4.90
Pancreatic lipase-related protein	*PLRP*	−3.48
SLC4-like anion exchanger	*AE*	2.24
Adenylyl cyclase 35C, isoform A	*AC35*	2.81
Protein kinase C	*PKC*	3.48
Adenylate cyclase type 9	*AC9*	3.52
Adenylate cyclase	*AC*	3.73
RAB8A, member RAS oncogene family	*Rabs*	4.25

### The neuroactive ligand-receptor interaction pathway is associated with phenol stress

The neuroactive ligand-receptor interaction pathway, which comprises a collection of receptors located on the plasma membranes, is involved in the transduction of signals from the extracellular environment into cells [55]. The neuroactive ligand-receptor interaction pathway was significantly affected under phenol stress, and this pathway may be associated with physiological and neuronal functions. In addition, 89 genes that were upregulated by phenol stress, and 83 genes that were downregulated by phenol stress, are involved in this pathway, as determined by KEGG analysis. Among these genes, 21 genes encoding receptors were significantly affected by phenol stress (Table 7). The neuroactive ligand-receptor interaction pathway can be divided into four subclasses according to the ligand structures, including class A (rhodopsin-like), class B (secretin-like), class C (metabotropic glutamate/pheromone) and channels/other receptors [56]. Phenol upregulated the expression of genes encoding class A amide receptors include the following: *FMRFar, Str2, Str1, Npra11 and Npra16*. In addition, phenol affected the expression of four genes encoding class B receptors, including *Relar2, Calcr, Oxr2* and *Dhr*. Most genes encoding receptors that were affected by phenol were in “channels or other receptors” category, including *Narβ3, Narβ2, Narα9, Narα11, Mgaba1, Ara2a, NMDAr, GPCR, GABAr, Tr21, Nara34e*, *Narβ3, Narβ2, Narα9*, *Narα11*, *Mgaba1, Ara2a, NMDAr, GPCR, GABAr, Tr21* and *Nara34e*.

**Table 7:** Genes in neuroactive ligand-receptor interaction pathways.

Description	Gene symbol	Ratio
Neuronal acetylcholine receptor subunit beta-3	*Narβ3*	−3952.57
Relaxin receptor 2	*Relar2*	−3831.42
Nicotinic acetylcholine receptor, beta-2 subunit	*Narβ2*	−50.56
Nicotinic acetylcholine receptor alpha 9 subunit	*Narα9*	−38.17
Nicotinic acetylcholine receptor subunit alpha 11	*Narα11*	−22.36
Coagulation factor VII	*F7*	−5.88
Coagulation factor X	*F5*	−3.7
leucine-rich transmembrane protein	*Leutp*	−2.33
Calcitonin receptor	*Calcr*	2.11
Glutamate receptor	*Glur*	2.54
FMRF amide receptor	*FMRFar*	2.67
Metabotropic GABA-B receptor subtype 1	*Mgaba1*	2.76
Adenosine receptor A2a	*Ara2a*	2.93
NMDA receptor 1	*NMDAr*	3.05
G-protein coupled receptor	*GPCR*	3.53
Orexin receptor type 2	*Oxr2*	3.67
Neuropeptide receptor A11	*Npra11*	4.2
Diuretic hormone receptor	*Dhr*	4.23
GABA receptor	*GABAr*	4.47
Serotonin receptor 2, isoform B	*Str2*	4.63
A16 Neuropeptide receptor A16	*Npra16*	4.97
Serotonin receptor type 1	*Str1*	9.25
Toll-like receptor 21	*Tr21*	9.51
Nicotinic acetylcholine receptor alpha 34E, isoform F	*Nara34e*	16.97

Analysis of the transcriptome showed that phenol affects 238 pathways in chironomid larvae. Among these pathways, the neuroactive ligand-receptor interaction pathway is an attractive target for further study because multiple receptors on the plasma membranes associated with cell signaling are located in this pathway. Bioamine is an important neurotransmitter that interacts with receptors to regulate some important biological functions, i.e., physiological rhythms, internal secretion, emotion, learning and memory [57]. In the neuroactive ligand-receptor interaction pathway, the nicotinic acetylcholine receptors were significantly affected by phenol. Nicotinic acetylcholine receptors (nAChRs) are cholinergic receptors that form ligand-gated ion channels in the plasma membranes of certain neurons and on the postsynaptic side of the neuromuscular junction. In insect nervous systems, nAChRs are the targets of insecticides such as neonicotinoid insecticides [58]–[59]. Under phenol stress, four genes encoding nAChRs subunits, including *Narβ3, Narβ2, Narα9* and *Narα11*, were downregulated, while *Nara34e* was upregulated, which demonstrates that phenol can alter nervous-related functions.Therefore, when harmful side effects of phenol are triggered in aquatic organisms, the neuroactive ligand-receptor interaction pathway may play important regulatory roles. However, further research using microarray and PCR technology is needed to confirm these results.

## Supporting Information

Figure S1 **Size distribution of Solexa sequencing contigs in** ***C. kiiensis*** **transcriptome.**
(TIF)

Table S1 **Top BLAST hits from NCBI nr database.** BLAST results against the NCBI nr database for all the distinct sequences with a cut-off Evalue above 10^−5^ are shown.
(XLS)

Table S2 **List of DEGs changed for 20 fold and more in PT library.**
(XLS)

Table S3 **Pathways enrichment for up-regulated and down-regulated DEGs.**
(DOC)
