# Supplementary figures and images for: Transcriptome Profiling of Chironomus kiinensis under Phenol Stress Using Solexa Sequencing Technology
Source: PLoS One. 2013 Mar 20;8(3):e58914. doi: 10.1371/journal.pone.0058914 (PMC3604134; doi:10.1371/journal.pone.0058914)

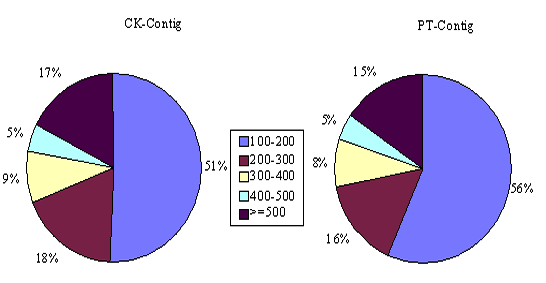

Supplement: Figure S1 — Size distribution of Solexa sequencing contigs in C. kiiensis transcriptome. (TIF) [file pone.0058914.s001.tif]
