# Supplementary material for: Transcriptome Profiling of Chironomus kiinensis under Phenol Stress Using Solexa Sequencing Technology
Source: PLoS One. 2013 Mar 20;8(3):e58914. doi: 10.1371/journal.pone.0058914 (PMC3604134; doi:10.1371/journal.pone.0058914)
Supplement: Table S3 — Pathways enrichment for up-regulated and down-regulated DEGs. (DOC) [file pone.0058914.s004.doc]

**Table S3. Pathways enrichment for up-regulated and down-regulated DEGs**

| **#** | **Pathway** | **Upregulated DEGs tested**  **（5091）** | **Downregulated DEGs tested**  **（3055）** | **Pvalue** | **Qvalue** | **Pathway ID** |
| --- | --- | --- | --- | --- | --- | --- |
| 1 | Pancreatic secretion | 87（1.71%） | 122（3.99%） | 3.45E-26 | 8.20E-24 | ko04972 |
| 2 | Protein digestion and absorption | 90（1.77%） | 101（3.31%） | 8.56E-20 | 1.02E-17 | ko04974 |
| 3 | Neuroactive ligand-receptor interaction | 89（1.75%） | 83（2.72%） | 5.40E-17 | 4.28E-15 | ko04080 |
| 4 | Influenza A | 83（1.63%） | 81（2.65%） | 7.27E-15 | 4.33E-13 | ko05164 |
| 5 | Complement and coagulation cascades | 33（0.65%） | 78（2.55%） | 2.02E-14 | 9.61E-13 | ko04610 |
| 6 | [Insect hormone biosynthesis](../../../../C:%5CDocuments%20and%20Settings%5CChuanwang%20CAO%5C%E6%A1%8C%E9%9D%A2%5C%E6%91%87%E8%9A%8A%E8%BD%AC%E5%BD%95%E6%9C%AC%E6%95%B0%E6%8D%AE%E7%BB%93%E6%9E%9C%E6%95%B4%E7%90%860225%5CCK-vs-PT.upregulation%20invovled%20in%20pathway.xls#RANGE!gene6%23RANGE!gene6) | 12（0.24%） | 21（0.69%） | 2.44E-08 | 9.70E-07 | ko00981 |
| 7 | [Pentose and glucuronate interconversions](../../../../C:%5CDocuments%20and%20Settings%5CChuanwang%20CAO%5C%E6%A1%8C%E9%9D%A2%5C%E6%91%87%E8%9A%8A%E8%BD%AC%E5%BD%95%E6%9C%AC%E6%95%B0%E6%8D%AE%E7%BB%93%E6%9E%9C%E6%95%B4%E7%90%860225%5CCK-vs-PT.upregulation%20invovled%20in%20pathway.xls#RANGE!gene7%23RANGE!gene7) | 40（0.79%） | 11（0.36%） | 5.58E-08 | 1.90E-06 | ko00040 |
| 8 | [ECM-receptor interaction](../../../../C:%5CDocuments%20and%20Settings%5CChuanwang%20CAO%5C%E6%A1%8C%E9%9D%A2%5C%E6%91%87%E8%9A%8A%E8%BD%AC%E5%BD%95%E6%9C%AC%E6%95%B0%E6%8D%AE%E7%BB%93%E6%9E%9C%E6%95%B4%E7%90%860225%5CCK-vs-PT.upregulation%20invovled%20in%20pathway.xls#RANGE!gene8%23RANGE!gene8) | 56（1.10%） | 54（1.77%） | 1.35E-07 | 4.02E-06 | ko04512 |
| 9 | [Hematopoietic cell lineage](../../../../C:%5CDocuments%20and%20Settings%5CChuanwang%20CAO%5C%E6%A1%8C%E9%9D%A2%5C%E6%91%87%E8%9A%8A%E8%BD%AC%E5%BD%95%E6%9C%AC%E6%95%B0%E6%8D%AE%E7%BB%93%E6%9E%9C%E6%95%B4%E7%90%860225%5CCK-vs-PT.upregulation%20invovled%20in%20pathway.xls#RANGE!gene9%23RANGE!gene9) | 34（0.67%） | 41（1.34%） | 2.13E-07 | 5.64E-06 | ko04640 |
| 10 | [Vitamin digestion and absorption](../../../../C:%5CDocuments%20and%20Settings%5CChuanwang%20CAO%5C%E6%A1%8C%E9%9D%A2%5C%E6%91%87%E8%9A%8A%E8%BD%AC%E5%BD%95%E6%9C%AC%E6%95%B0%E6%8D%AE%E7%BB%93%E6%9E%9C%E6%95%B4%E7%90%860225%5CCK-vs-PT.upregulation%20invovled%20in%20pathway.xls#RANGE!gene10%23RANGE!gene10) | 30（0.59%） | 25（0.82%） | 4.29E-07 | 1.02E-05 | ko04977 |
| 11 | [Amoebiasis](../../../../C:%5CDocuments%20and%20Settings%5CChuanwang%20CAO%5C%E6%A1%8C%E9%9D%A2%5C%E6%91%87%E8%9A%8A%E8%BD%AC%E5%BD%95%E6%9C%AC%E6%95%B0%E6%8D%AE%E7%BB%93%E6%9E%9C%E6%95%B4%E7%90%860225%5CCK-vs-PT.upregulation%20invovled%20in%20pathway.xls#RANGE!gene11%23RANGE!gene11) | 49（0.96%） | 67（2.19%） | 9.01E-07 | 1.95E-05 | ko05146 |
| 12 | [Metabolic pathways](../../../../C:%5CDocuments%20and%20Settings%5CChuanwang%20CAO%5C%E6%A1%8C%E9%9D%A2%5C%E6%91%87%E8%9A%8A%E8%BD%AC%E5%BD%95%E6%9C%AC%E6%95%B0%E6%8D%AE%E7%BB%93%E6%9E%9C%E6%95%B4%E7%90%860225%5CCK-vs-PT.upregulation%20invovled%20in%20pathway.xls#RANGE!gene12%23RANGE!gene12) | 336（6.60%） | 283（9.26%） | 9.85E-07 | 1.95E-05 | ko01100 |
| 13 | [Metabolism of xenobiotics by cytochrome P450](../../../../C:%5CDocuments%20and%20Settings%5CChuanwang%20CAO%5C%E6%A1%8C%E9%9D%A2%5C%E6%91%87%E8%9A%8A%E8%BD%AC%E5%BD%95%E6%9C%AC%E6%95%B0%E6%8D%AE%E7%BB%93%E6%9E%9C%E6%95%B4%E7%90%860225%5CCK-vs-PT.upregulation%20invovled%20in%20pathway.xls#RANGE!gene13%23RANGE!gene13) | 48（0.94%） | 16（0.52%） | 1.56E-06 | 2.85E-05 | ko00980 |
| 14 | [alpha-Linolenic acid metabolism](../../../../C:%5CDocuments%20and%20Settings%5CChuanwang%20CAO%5C%E6%A1%8C%E9%9D%A2%5C%E6%91%87%E8%9A%8A%E8%BD%AC%E5%BD%95%E6%9C%AC%E6%95%B0%E6%8D%AE%E7%BB%93%E6%9E%9C%E6%95%B4%E7%90%860225%5CCK-vs-PT.upregulation%20invovled%20in%20pathway.xls#RANGE!gene14%23RANGE!gene14) | 9（0.18%） | 28（0.92%） | 4.03E-06 | 6.84E-05 | ko00592 |
| 15 | [Drug metabolism - cytochrome P450](../../../../C:%5CDocuments%20and%20Settings%5CChuanwang%20CAO%5C%E6%A1%8C%E9%9D%A2%5C%E6%91%87%E8%9A%8A%E8%BD%AC%E5%BD%95%E6%9C%AC%E6%95%B0%E6%8D%AE%E7%BB%93%E6%9E%9C%E6%95%B4%E7%90%860225%5CCK-vs-PT.upregulation%20invovled%20in%20pathway.xls#RANGE!gene15%23RANGE!gene15) | 48（0.94%） | 17（0.56%） | 1.04E-05 | 1.66E-04 | ko00982 |
| 16 | [Drug metabolism - other enzymes](../../../../C:%5CDocuments%20and%20Settings%5CChuanwang%20CAO%5C%E6%A1%8C%E9%9D%A2%5C%E6%91%87%E8%9A%8A%E8%BD%AC%E5%BD%95%E6%9C%AC%E6%95%B0%E6%8D%AE%E7%BB%93%E6%9E%9C%E6%95%B4%E7%90%860225%5CCK-vs-PT.upregulation%20invovled%20in%20pathway.xls#RANGE!gene16%23RANGE!gene16) | 44（0.86%） | 30（0.98%） | 2.11E-05 | 3.13E-04 | ko00983 |
| 17 | [Toll-like receptor signaling pathway](../../../../C:%5CDocuments%20and%20Settings%5CChuanwang%20CAO%5C%E6%A1%8C%E9%9D%A2%5C%E6%91%87%E8%9A%8A%E8%BD%AC%E5%BD%95%E6%9C%AC%E6%95%B0%E6%8D%AE%E7%BB%93%E6%9E%9C%E6%95%B4%E7%90%860225%5CCK-vs-PT.upregulation%20invovled%20in%20pathway.xls#RANGE!gene17%23RANGE!gene17) | 32（0.63%） | 21（0.69%） | 2.53E-05 | 3.54E-04 | ko04620 |
| 18 | [Glutathione metabolism](../../../../C:%5CDocuments%20and%20Settings%5CChuanwang%20CAO%5C%E6%A1%8C%E9%9D%A2%5C%E6%91%87%E8%9A%8A%E8%BD%AC%E5%BD%95%E6%9C%AC%E6%95%B0%E6%8D%AE%E7%BB%93%E6%9E%9C%E6%95%B4%E7%90%860225%5CCK-vs-PT.upregulation%20invovled%20in%20pathway.xls#RANGE!gene18%23RANGE!gene18) | 38（0.75%） | 32（1.05%） | 3.11E-05 | 4.12E-04 | ko00480 |
| 19 | [Vibrio cholerae infection](../../../../C:%5CDocuments%20and%20Settings%5CChuanwang%20CAO%5C%E6%A1%8C%E9%9D%A2%5C%E6%91%87%E8%9A%8A%E8%BD%AC%E5%BD%95%E6%9C%AC%E6%95%B0%E6%8D%AE%E7%BB%93%E6%9E%9C%E6%95%B4%E7%90%860225%5CCK-vs-PT.upregulation%20invovled%20in%20pathway.xls#RANGE!gene19%23RANGE!gene19) | 42（0.82%） | 56（1.83%） | 3.78E-05 | 4.74E-04 | ko05110 |
| 20 | [Bile secretion](../../../../C:%5CDocuments%20and%20Settings%5CChuanwang%20CAO%5C%E6%A1%8C%E9%9D%A2%5C%E6%91%87%E8%9A%8A%E8%BD%AC%E5%BD%95%E6%9C%AC%E6%95%B0%E6%8D%AE%E7%BB%93%E6%9E%9C%E6%95%B4%E7%90%860225%5CCK-vs-PT.upregulation%20invovled%20in%20pathway.xls#RANGE!gene20%23RANGE!gene20) | 68（1.34%） | 17（0.56%） | 4.17E-05 | 4.96E-04 | ko04976 |
| 21 | [Nitrogen metabolism](../../../../C:%5CDocuments%20and%20Settings%5CChuanwang%20CAO%5C%E6%A1%8C%E9%9D%A2%5C%E6%91%87%E8%9A%8A%E8%BD%AC%E5%BD%95%E6%9C%AC%E6%95%B0%E6%8D%AE%E7%BB%93%E6%9E%9C%E6%95%B4%E7%90%860225%5CCK-vs-PT.upregulation%20invovled%20in%20pathway.xls#RANGE!gene21%23RANGE!gene21) | 10（0.20%） | 11（0.36%） | 5.47E-05 | 6.11E-04 | ko00910 |
| 22 | [Ascorbate and aldarate metabolism](../../../../C:%5CDocuments%20and%20Settings%5CChuanwang%20CAO%5C%E6%A1%8C%E9%9D%A2%5C%E6%91%87%E8%9A%8A%E8%BD%AC%E5%BD%95%E6%9C%AC%E6%95%B0%E6%8D%AE%E7%BB%93%E6%9E%9C%E6%95%B4%E7%90%860225%5CCK-vs-PT.upregulation%20invovled%20in%20pathway.xls#RANGE!gene22%23RANGE!gene22) | 28（0.55%） | 9（0.29%） | 5.65E-05 | 6.11E-04 | ko00053 |
| 23 | [Glycine, serine and threonine metabolism](../../../../C:%5CDocuments%20and%20Settings%5CChuanwang%20CAO%5C%E6%A1%8C%E9%9D%A2%5C%E6%91%87%E8%9A%8A%E8%BD%AC%E5%BD%95%E6%9C%AC%E6%95%B0%E6%8D%AE%E7%BB%93%E6%9E%9C%E6%95%B4%E7%90%860225%5CCK-vs-PT.upregulation%20invovled%20in%20pathway.xls#RANGE!gene23%23RANGE!gene23) | 16（0.31%） | 26（0.85%） | 6.91E-05 | 7.15E-04 | ko00260 |
| 24 | [Porphyrin and chlorophyll metabolism](../../../../C:%5CDocuments%20and%20Settings%5CChuanwang%20CAO%5C%E6%A1%8C%E9%9D%A2%5C%E6%91%87%E8%9A%8A%E8%BD%AC%E5%BD%95%E6%9C%AC%E6%95%B0%E6%8D%AE%E7%BB%93%E6%9E%9C%E6%95%B4%E7%90%860225%5CCK-vs-PT.upregulation%20invovled%20in%20pathway.xls#RANGE!gene24%23RANGE!gene24) | 30（0.59%） | 9（0.29%） | 7.78E-05 | 7.67E-04 | ko00860 |
| 25 | [Staphylococcus aureus infection](../../../../C:%5CDocuments%20and%20Settings%5CChuanwang%20CAO%5C%E6%A1%8C%E9%9D%A2%5C%E6%91%87%E8%9A%8A%E8%BD%AC%E5%BD%95%E6%9C%AC%E6%95%B0%E6%8D%AE%E7%BB%93%E6%9E%9C%E6%95%B4%E7%90%860225%5CCK-vs-PT.upregulation%20invovled%20in%20pathway.xls#RANGE!gene25%23RANGE!gene25) | 13（0.26%） | 27（0.88%） | 8.06E-05 | 7.67E-04 | ko05150 |
| 26 | [PPAR signaling pathway](../../../../C:%5CDocuments%20and%20Settings%5CChuanwang%20CAO%5C%E6%A1%8C%E9%9D%A2%5C%E6%91%87%E8%9A%8A%E8%BD%AC%E5%BD%95%E6%9C%AC%E6%95%B0%E6%8D%AE%E7%BB%93%E6%9E%9C%E6%95%B4%E7%90%860225%5CCK-vs-PT.upregulation%20invovled%20in%20pathway.xls#RANGE!gene26%23RANGE!gene26) | 25（0.49%） | 38（1.24%） | 0.000106 | 9.67E-04 | ko03320 |
| 27 | [Glycerolipid metabolism](../../../../C:%5CDocuments%20and%20Settings%5CChuanwang%20CAO%5C%E6%A1%8C%E9%9D%A2%5C%E6%91%87%E8%9A%8A%E8%BD%AC%E5%BD%95%E6%9C%AC%E6%95%B0%E6%8D%AE%E7%BB%93%E6%9E%9C%E6%95%B4%E7%90%860225%5CCK-vs-PT.upregulation%20invovled%20in%20pathway.xls#RANGE!gene27%23RANGE!gene27) | 24（0.47%） | 32（1.05%） | 0.000307 | 2.71E-03 | ko00561 |
| 28 | [Cysteine and methionine metabolism](../../../../C:%5CDocuments%20and%20Settings%5CChuanwang%20CAO%5C%E6%A1%8C%E9%9D%A2%5C%E6%91%87%E8%9A%8A%E8%BD%AC%E5%BD%95%E6%9C%AC%E6%95%B0%E6%8D%AE%E7%BB%93%E6%9E%9C%E6%95%B4%E7%90%860225%5CCK-vs-PT.upregulation%20invovled%20in%20pathway.xls#RANGE!gene28%23RANGE!gene28) | 18（0.35%） | 19（0.62%） | 0.000389 | 3.30E-03 | ko00270 |
| 29 | [Valine, leucine and isoleucine biosynthesis](../../../../C:%5CDocuments%20and%20Settings%5CChuanwang%20CAO%5C%E6%A1%8C%E9%9D%A2%5C%E6%91%87%E8%9A%8A%E8%BD%AC%E5%BD%95%E6%9C%AC%E6%95%B0%E6%8D%AE%E7%BB%93%E6%9E%9C%E6%95%B4%E7%90%860225%5CCK-vs-PT.upregulation%20invovled%20in%20pathway.xls#RANGE!gene29%23RANGE!gene29) | 13（0.26%） | 11（0.36%） | 0.000966 | 7.93E-03 | ko00290 |
| 30 | [Fat digestion and absorption](../../../../C:%5CDocuments%20and%20Settings%5CChuanwang%20CAO%5C%E6%A1%8C%E9%9D%A2%5C%E6%91%87%E8%9A%8A%E8%BD%AC%E5%BD%95%E6%9C%AC%E6%95%B0%E6%8D%AE%E7%BB%93%E6%9E%9C%E6%95%B4%E7%90%860225%5CCK-vs-PT.upregulation%20invovled%20in%20pathway.xls#RANGE!gene30%23RANGE!gene30) | 16（0.31%） | 28（0.92%） | 0.001188 | 9.43E-03 | ko04975 |
| 31 | [RNA polymerase](../../../../C:%5CDocuments%20and%20Settings%5CChuanwang%20CAO%5C%E6%A1%8C%E9%9D%A2%5C%E6%91%87%E8%9A%8A%E8%BD%AC%E5%BD%95%E6%9C%AC%E6%95%B0%E6%8D%AE%E7%BB%93%E6%9E%9C%E6%95%B4%E7%90%860225%5CCK-vs-PT.upregulation%20invovled%20in%20pathway.xls#RANGE!gene31%23RANGE!gene31) | 47（0.92%） | 8（0.26%） | 0.001262 | 9.47E-03 | ko03020 |
| 32 | [Folate biosynthesis](../../../../C:%5CDocuments%20and%20Settings%5CChuanwang%20CAO%5C%E6%A1%8C%E9%9D%A2%5C%E6%91%87%E8%9A%8A%E8%BD%AC%E5%BD%95%E6%9C%AC%E6%95%B0%E6%8D%AE%E7%BB%93%E6%9E%9C%E6%95%B4%E7%90%860225%5CCK-vs-PT.upregulation%20invovled%20in%20pathway.xls#RANGE!gene32%23RANGE!gene32) | 7（0.14%） | 11（0.36%） | 0.001273 | 9.47E-03 | ko00790 |
| 33 | [Steroid hormone biosynthesis](../../../../C:%5CDocuments%20and%20Settings%5CChuanwang%20CAO%5C%E6%A1%8C%E9%9D%A2%5C%E6%91%87%E8%9A%8A%E8%BD%AC%E5%BD%95%E6%9C%AC%E6%95%B0%E6%8D%AE%E7%BB%93%E6%9E%9C%E6%95%B4%E7%90%860225%5CCK-vs-PT.upregulation%20invovled%20in%20pathway.xls#RANGE!gene33%23RANGE!gene33) | 32（0.63%） | 13（0.43%） | 0.001341 | 9.67E-03 | ko00140 |
| 34 | [Pyruvate metabolism](../../../../C:%5CDocuments%20and%20Settings%5CChuanwang%20CAO%5C%E6%A1%8C%E9%9D%A2%5C%E6%91%87%E8%9A%8A%E8%BD%AC%E5%BD%95%E6%9C%AC%E6%95%B0%E6%8D%AE%E7%BB%93%E6%9E%9C%E6%95%B4%E7%90%860225%5CCK-vs-PT.upregulation%20invovled%20in%20pathway.xls#RANGE!gene34%23RANGE!gene34) | 26（0.51%） | 8（0.26%） | 0.00142 | 9.94E-03 | ko00620 |
| 35 | [Other types of O-glycan biosynthesis](../../../../C:%5CDocuments%20and%20Settings%5CChuanwang%20CAO%5C%E6%A1%8C%E9%9D%A2%5C%E6%91%87%E8%9A%8A%E8%BD%AC%E5%BD%95%E6%9C%AC%E6%95%B0%E6%8D%AE%E7%BB%93%E6%9E%9C%E6%95%B4%E7%90%860225%5CCK-vs-PT.upregulation%20invovled%20in%20pathway.xls#RANGE!gene35%23RANGE!gene35) | 28（0.55%） | 9（0.29%） | 0.001619 | 1.10E-02 | ko00514 |
| 36 | [Pertussis](../../../../C:%5CDocuments%20and%20Settings%5CChuanwang%20CAO%5C%E6%A1%8C%E9%9D%A2%5C%E6%91%87%E8%9A%8A%E8%BD%AC%E5%BD%95%E6%9C%AC%E6%95%B0%E6%8D%AE%E7%BB%93%E6%9E%9C%E6%95%B4%E7%90%860225%5CCK-vs-PT.upregulation%20invovled%20in%20pathway.xls#RANGE!gene36%23RANGE!gene36) | 22（0.43%） | 12（0.39%） | 0.002005 | 1.31E-02 | ko05133 |
| 37 | [Starch and sucrose metabolism](../../../../C:%5CDocuments%20and%20Settings%5CChuanwang%20CAO%5C%E6%A1%8C%E9%9D%A2%5C%E6%91%87%E8%9A%8A%E8%BD%AC%E5%BD%95%E6%9C%AC%E6%95%B0%E6%8D%AE%E7%BB%93%E6%9E%9C%E6%95%B4%E7%90%860225%5CCK-vs-PT.upregulation%20invovled%20in%20pathway.xls#RANGE!gene37%23RANGE!gene37) | 32（0.63%） | 17（0.56%） | 0.002043 | 1.31E-02 | ko00500 |
| 38 | [Ubiquinone and other terpenoid-quinone biosynthesis](../../../../C:%5CDocuments%20and%20Settings%5CChuanwang%20CAO%5C%E6%A1%8C%E9%9D%A2%5C%E6%91%87%E8%9A%8A%E8%BD%AC%E5%BD%95%E6%9C%AC%E6%95%B0%E6%8D%AE%E7%BB%93%E6%9E%9C%E6%95%B4%E7%90%860225%5CCK-vs-PT.upregulation%20invovled%20in%20pathway.xls#RANGE!gene38%23RANGE!gene38) | 7（0.14%） | 17（0.56%） | 0.002395 | 1.50E-02 | ko00130 |
| 39 | [Tyrosine metabolism](../../../../C:%5CDocuments%20and%20Settings%5CChuanwang%20CAO%5C%E6%A1%8C%E9%9D%A2%5C%E6%91%87%E8%9A%8A%E8%BD%AC%E5%BD%95%E6%9C%AC%E6%95%B0%E6%8D%AE%E7%BB%93%E6%9E%9C%E6%95%B4%E7%90%860225%5CCK-vs-PT.upregulation%20invovled%20in%20pathway.xls#RANGE!gene39%23RANGE!gene39) | 25（0.49%） | 31（1.01%） | 0.002836 | 1.73E-02 | ko00350 |
| 40 | [Retinol metabolism](../../../../C:%5CDocuments%20and%20Settings%5CChuanwang%20CAO%5C%E6%A1%8C%E9%9D%A2%5C%E6%91%87%E8%9A%8A%E8%BD%AC%E5%BD%95%E6%9C%AC%E6%95%B0%E6%8D%AE%E7%BB%93%E6%9E%9C%E6%95%B4%E7%90%860225%5CCK-vs-PT.upregulation%20invovled%20in%20pathway.xls#RANGE!gene40%23RANGE!gene40) | 37（0.73%） | 14（0.46%） | 0.003953 | 2.35E-02 | ko00830 |
| 41 | [Focal adhesion](../../../../C:%5CDocuments%20and%20Settings%5CChuanwang%20CAO%5C%E6%A1%8C%E9%9D%A2%5C%E6%91%87%E8%9A%8A%E8%BD%AC%E5%BD%95%E6%9C%AC%E6%95%B0%E6%8D%AE%E7%BB%93%E6%9E%9C%E6%95%B4%E7%90%860225%5CCK-vs-PT.upregulation%20invovled%20in%20pathway.xls#RANGE!gene41%23RANGE!gene41) | 91（1.79%） | 55（1.80%） | 0.004963 | 2.88E-02 | ko04510 |
| 42 | [Phenylalanine metabolism](../../../../C:%5CDocuments%20and%20Settings%5CChuanwang%20CAO%5C%E6%A1%8C%E9%9D%A2%5C%E6%91%87%E8%9A%8A%E8%BD%AC%E5%BD%95%E6%9C%AC%E6%95%B0%E6%8D%AE%E7%BB%93%E6%9E%9C%E6%95%B4%E7%90%860225%5CCK-vs-PT.upregulation%20invovled%20in%20pathway.xls#RANGE!gene42%23RANGE!gene42) | 7（0.14%） | 19（0.62%） | 0.005248 | 2.97E-02 | ko00360 |
| 43 | [Lysosome](../../../../C:%5CDocuments%20and%20Settings%5CChuanwang%20CAO%5C%E6%A1%8C%E9%9D%A2%5C%E6%91%87%E8%9A%8A%E8%BD%AC%E5%BD%95%E6%9C%AC%E6%95%B0%E6%8D%AE%E7%BB%93%E6%9E%9C%E6%95%B4%E7%90%860225%5CCK-vs-PT.upregulation%20invovled%20in%20pathway.xls#RANGE!gene43%23RANGE!gene43) | 46（0.90%） | 41（1.34%） | 0.005643 | 3.12E-02 | ko04142 |
| 44 | [Autoimmune thyroid disease](../../../../C:%5CDocuments%20and%20Settings%5CChuanwang%20CAO%5C%E6%A1%8C%E9%9D%A2%5C%E6%91%87%E8%9A%8A%E8%BD%AC%E5%BD%95%E6%9C%AC%E6%95%B0%E6%8D%AE%E7%BB%93%E6%9E%9C%E6%95%B4%E7%90%860225%5CCK-vs-PT.upregulation%20invovled%20in%20pathway.xls#RANGE!gene44%23RANGE!gene44) | 2（0.04%） | 7（0.23%） | 0.007323 | 3.96E-02 | ko05320 |
| 45 | [Amino sugar and nucleotide sugar metabolism](../../../../C:%5CDocuments%20and%20Settings%5CChuanwang%20CAO%5C%E6%A1%8C%E9%9D%A2%5C%E6%91%87%E8%9A%8A%E8%BD%AC%E5%BD%95%E6%9C%AC%E6%95%B0%E6%8D%AE%E7%BB%93%E6%9E%9C%E6%95%B4%E7%90%860225%5CCK-vs-PT.upregulation%20invovled%20in%20pathway.xls#RANGE!gene45%23RANGE!gene45) | 22（0.43%） | 26（0.85%） | 0.007949 | 4.20E-02 | ko00520 |
| 46 | [Osteoclast differentiation](../../../../C:%5CDocuments%20and%20Settings%5CChuanwang%20CAO%5C%E6%A1%8C%E9%9D%A2%5C%E6%91%87%E8%9A%8A%E8%BD%AC%E5%BD%95%E6%9C%AC%E6%95%B0%E6%8D%AE%E7%BB%93%E6%9E%9C%E6%95%B4%E7%90%860225%5CCK-vs-PT.upregulation%20invovled%20in%20pathway.xls#RANGE!gene46%23RANGE!gene46) | 24（0.47%） | 6（0.20%） | 0.010469 | 5.42E-02 | ko04380 |
| 47 | [Galactose metabolism](../../../../C:%5CDocuments%20and%20Settings%5CChuanwang%20CAO%5C%E6%A1%8C%E9%9D%A2%5C%E6%91%87%E8%9A%8A%E8%BD%AC%E5%BD%95%E6%9C%AC%E6%95%B0%E6%8D%AE%E7%BB%93%E6%9E%9C%E6%95%B4%E7%90%860225%5CCK-vs-PT.upregulation%20invovled%20in%20pathway.xls#RANGE!gene47%23RANGE!gene47) | 16（0.31%） | 12（0.39%） | 0.012947 | 6.50E-02 | ko00052 |
| 48 | [Renin-angiotensin system](../../../../C:%5CDocuments%20and%20Settings%5CChuanwang%20CAO%5C%E6%A1%8C%E9%9D%A2%5C%E6%91%87%E8%9A%8A%E8%BD%AC%E5%BD%95%E6%9C%AC%E6%95%B0%E6%8D%AE%E7%BB%93%E6%9E%9C%E6%95%B4%E7%90%860225%5CCK-vs-PT.upregulation%20invovled%20in%20pathway.xls#RANGE!gene48%23RANGE!gene48) | 14（0.27%） | 20（0.65%） | 0.013307 | 6.50E-02 | ko04614 |
| 49 | [Glycerophospholipid metabolism](../../../../C:%5CDocuments%20and%20Settings%5CChuanwang%20CAO%5C%E6%A1%8C%E9%9D%A2%5C%E6%91%87%E8%9A%8A%E8%BD%AC%E5%BD%95%E6%9C%AC%E6%95%B0%E6%8D%AE%E7%BB%93%E6%9E%9C%E6%95%B4%E7%90%860225%5CCK-vs-PT.upregulation%20invovled%20in%20pathway.xls#RANGE!gene49%23RANGE!gene49) | 33（0.65%） | 33（1.08%） | 0.013466 | 6.50E-02 | ko00564 |
| 50 | [Peroxisome](../../../../C:%5CDocuments%20and%20Settings%5CChuanwang%20CAO%5C%E6%A1%8C%E9%9D%A2%5C%E6%91%87%E8%9A%8A%E8%BD%AC%E5%BD%95%E6%9C%AC%E6%95%B0%E6%8D%AE%E7%BB%93%E6%9E%9C%E6%95%B4%E7%90%860225%5CCK-vs-PT.upregulation%20invovled%20in%20pathway.xls#RANGE!gene50%23RANGE!gene50) | 24（0.47%） | 43（1.41%） | 0.013901 | 6.50E-02 | ko04146 |
| 51 | [Arachidonic acid metabolism](../../../../C:%5CDocuments%20and%20Settings%5CChuanwang%20CAO%5C%E6%A1%8C%E9%9D%A2%5C%E6%91%87%E8%9A%8A%E8%BD%AC%E5%BD%95%E6%9C%AC%E6%95%B0%E6%8D%AE%E7%BB%93%E6%9E%9C%E6%95%B4%E7%90%860225%5CCK-vs-PT.upregulation%20invovled%20in%20pathway.xls#RANGE!gene51%23RANGE!gene51) | 9（0.18%） | 9（0.29%） | 0.013923 | 6.50E-02 | ko00590 |
| 52 | [Phagosome](../../../../C:%5CDocuments%20and%20Settings%5CChuanwang%20CAO%5C%E6%A1%8C%E9%9D%A2%5C%E6%91%87%E8%9A%8A%E8%BD%AC%E5%BD%95%E6%9C%AC%E6%95%B0%E6%8D%AE%E7%BB%93%E6%9E%9C%E6%95%B4%E7%90%860225%5CCK-vs-PT.upregulation%20invovled%20in%20pathway.xls#RANGE!gene52%23RANGE!gene52) | 47（0.92%） | 48（1.57%） | 0.015065 | 6.90E-02 | ko04145 |
| 53 | [Axon guidance](../../../../C:%5CDocuments%20and%20Settings%5CChuanwang%20CAO%5C%E6%A1%8C%E9%9D%A2%5C%E6%91%87%E8%9A%8A%E8%BD%AC%E5%BD%95%E6%9C%AC%E6%95%B0%E6%8D%AE%E7%BB%93%E6%9E%9C%E6%95%B4%E7%90%860225%5CCK-vs-PT.upregulation%20invovled%20in%20pathway.xls#RANGE!gene53%23RANGE!gene53) | 56（1.10%） | 23（0.75%） | 0.017641 | 7.92E-02 | ko04360 |
| 54 | [Riboflavin metabolism](../../../../C:%5CDocuments%20and%20Settings%5CChuanwang%20CAO%5C%E6%A1%8C%E9%9D%A2%5C%E6%91%87%E8%9A%8A%E8%BD%AC%E5%BD%95%E6%9C%AC%E6%95%B0%E6%8D%AE%E7%BB%93%E6%9E%9C%E6%95%B4%E7%90%860225%5CCK-vs-PT.upregulation%20invovled%20in%20pathway.xls#RANGE!gene54%23RANGE!gene54) | 2（0.04%） | 7（0.23%） | 0.020622 | 9.09E-02 | ko00740 |
| 55 | [Leishmaniasis](../../../../C:%5CDocuments%20and%20Settings%5CChuanwang%20CAO%5C%E6%A1%8C%E9%9D%A2%5C%E6%91%87%E8%9A%8A%E8%BD%AC%E5%BD%95%E6%9C%AC%E6%95%B0%E6%8D%AE%E7%BB%93%E6%9E%9C%E6%95%B4%E7%90%860225%5CCK-vs-PT.upregulation%20invovled%20in%20pathway.xls#RANGE!gene55%23RANGE!gene55) | 18（0.35%） | 8（0.26%） | 0.021197 | 9.17E-02 | ko05140 |
| 56 | [Hepatitis C](../../../../C:%5CDocuments%20and%20Settings%5CChuanwang%20CAO%5C%E6%A1%8C%E9%9D%A2%5C%E6%91%87%E8%9A%8A%E8%BD%AC%E5%BD%95%E6%9C%AC%E6%95%B0%E6%8D%AE%E7%BB%93%E6%9E%9C%E6%95%B4%E7%90%860225%5CCK-vs-PT.upregulation%20invovled%20in%20pathway.xls#RANGE!gene56%23RANGE!gene56) | 23（0.45%） | 13（0.43%） | 0.026874 | 1.14E-01 | ko05160 |
| 57 | [Asthma](../../../../C:%5CDocuments%20and%20Settings%5CChuanwang%20CAO%5C%E6%A1%8C%E9%9D%A2%5C%E6%91%87%E8%9A%8A%E8%BD%AC%E5%BD%95%E6%9C%AC%E6%95%B0%E6%8D%AE%E7%BB%93%E6%9E%9C%E6%95%B4%E7%90%860225%5CCK-vs-PT.upregulation%20invovled%20in%20pathway.xls#RANGE!gene57%23RANGE!gene57) | 0（0.00%） | 4（0.13%） | 0.028474 | 1.19E-01 | ko05310 |
| 58 | [Antigen processing and presentation](../../../../C:%5CDocuments%20and%20Settings%5CChuanwang%20CAO%5C%E6%A1%8C%E9%9D%A2%5C%E6%91%87%E8%9A%8A%E8%BD%AC%E5%BD%95%E6%9C%AC%E6%95%B0%E6%8D%AE%E7%BB%93%E6%9E%9C%E6%95%B4%E7%90%860225%5CCK-vs-PT.upregulation%20invovled%20in%20pathway.xls#RANGE!gene58%23RANGE!gene58) | 16（0.31%） | 11（0.36%） | 0.029831 | 1.22E-01 | ko04612 |
| 59 | [Allograft rejection](../../../../C:%5CDocuments%20and%20Settings%5CChuanwang%20CAO%5C%E6%A1%8C%E9%9D%A2%5C%E6%91%87%E8%9A%8A%E8%BD%AC%E5%BD%95%E6%9C%AC%E6%95%B0%E6%8D%AE%E7%BB%93%E6%9E%9C%E6%95%B4%E7%90%860225%5CCK-vs-PT.upregulation%20invovled%20in%20pathway.xls#RANGE!gene59%23RANGE!gene59) | 2（0.04%） | 0（0.00%） | 0.036516 | 1.45E-01 | ko05330 |
| 60 | [Graft-versus-host disease](../../../../C:%5CDocuments%20and%20Settings%5CChuanwang%20CAO%5C%E6%A1%8C%E9%9D%A2%5C%E6%91%87%E8%9A%8A%E8%BD%AC%E5%BD%95%E6%9C%AC%E6%95%B0%E6%8D%AE%E7%BB%93%E6%9E%9C%E6%95%B4%E7%90%860225%5CCK-vs-PT.upregulation%20invovled%20in%20pathway.xls#RANGE!gene60%23RANGE!gene60) | 2（0.04%） | 0（0.00%） | 0.036516 | 1.45E-01 | ko05332 |
| 61 | [Carbohydrate digestion and absorption](../../../../C:%5CDocuments%20and%20Settings%5CChuanwang%20CAO%5C%E6%A1%8C%E9%9D%A2%5C%E6%91%87%E8%9A%8A%E8%BD%AC%E5%BD%95%E6%9C%AC%E6%95%B0%E6%8D%AE%E7%BB%93%E6%9E%9C%E6%95%B4%E7%90%860225%5CCK-vs-PT.upregulation%20invovled%20in%20pathway.xls#RANGE!gene61%23RANGE!gene61) | 9（0.18%） | 16（0.52%） | 0.037051 | 1.45E-01 | ko04973 |
| 62 | [Long-term depression](../../../../C:%5CDocuments%20and%20Settings%5CChuanwang%20CAO%5C%E6%A1%8C%E9%9D%A2%5C%E6%91%87%E8%9A%8A%E8%BD%AC%E5%BD%95%E6%9C%AC%E6%95%B0%E6%8D%AE%E7%BB%93%E6%9E%9C%E6%95%B4%E7%90%860225%5CCK-vs-PT.upregulation%20invovled%20in%20pathway.xls#RANGE!gene62%23RANGE!gene62) | 23（0.45%） | 15（0.49%） | 0.039547 | 1.52E-01 | ko04730 |
| 63 | [Toxoplasmosis](../../../../C:%5CDocuments%20and%20Settings%5CChuanwang%20CAO%5C%E6%A1%8C%E9%9D%A2%5C%E6%91%87%E8%9A%8A%E8%BD%AC%E5%BD%95%E6%9C%AC%E6%95%B0%E6%8D%AE%E7%BB%93%E6%9E%9C%E6%95%B4%E7%90%860225%5CCK-vs-PT.upregulation%20invovled%20in%20pathway.xls#RANGE!gene63%23RANGE!gene63) | 30（0.59%） | 16（0.52%） | 0.047147 | 1.78E-01 | ko05145 |
| 64 | [Pathogenic Escherichia coli infection](../../../../C:%5CDocuments%20and%20Settings%5CChuanwang%20CAO%5C%E6%A1%8C%E9%9D%A2%5C%E6%91%87%E8%9A%8A%E8%BD%AC%E5%BD%95%E6%9C%AC%E6%95%B0%E6%8D%AE%E7%BB%93%E6%9E%9C%E6%95%B4%E7%90%860225%5CCK-vs-PT.upregulation%20invovled%20in%20pathway.xls#RANGE!gene64%23RANGE!gene64) | 41（0.81%） | 24（0.79%） | 0.048659 | 1.81E-01 | ko05130 |
| 65 | [Cyanoamino acid metabolism](../../../../C:%5CDocuments%20and%20Settings%5CChuanwang%20CAO%5C%E6%A1%8C%E9%9D%A2%5C%E6%91%87%E8%9A%8A%E8%BD%AC%E5%BD%95%E6%9C%AC%E6%95%B0%E6%8D%AE%E7%BB%93%E6%9E%9C%E6%95%B4%E7%90%860225%5CCK-vs-PT.upregulation%20invovled%20in%20pathway.xls#RANGE!gene65%23RANGE!gene65) | 1（0.02%） | 7（0.23%） | 0.056811 | 2.08E-01 | ko00460 |
| 66 | [Regulation of actin cytoskeleton](../../../../C:%5CDocuments%20and%20Settings%5CChuanwang%20CAO%5C%E6%A1%8C%E9%9D%A2%5C%E6%91%87%E8%9A%8A%E8%BD%AC%E5%BD%95%E6%9C%AC%E6%95%B0%E6%8D%AE%E7%BB%93%E6%9E%9C%E6%95%B4%E7%90%860225%5CCK-vs-PT.upregulation%20invovled%20in%20pathway.xls#RANGE!gene66%23RANGE!gene66) | 106（2.08%） | 42（1.37%） | 0.060563 | 2.18E-01 | ko04810 |
| 67 | [Proximal tubule bicarbonate reclamation](../../../../C:%5CDocuments%20and%20Settings%5CChuanwang%20CAO%5C%E6%A1%8C%E9%9D%A2%5C%E6%91%87%E8%9A%8A%E8%BD%AC%E5%BD%95%E6%9C%AC%E6%95%B0%E6%8D%AE%E7%BB%93%E6%9E%9C%E6%95%B4%E7%90%860225%5CCK-vs-PT.upregulation%20invovled%20in%20pathway.xls#RANGE!gene67%23RANGE!gene67) | 9（0.18%） | 6（0.20%） | 0.067885 | 2.41E-01 | ko04964 |
| 68 | [One carbon pool by folate](../../../../C:%5CDocuments%20and%20Settings%5CChuanwang%20CAO%5C%E6%A1%8C%E9%9D%A2%5C%E6%91%87%E8%9A%8A%E8%BD%AC%E5%BD%95%E6%9C%AC%E6%95%B0%E6%8D%AE%E7%BB%93%E6%9E%9C%E6%95%B4%E7%90%860225%5CCK-vs-PT.upregulation%20invovled%20in%20pathway.xls#RANGE!gene68%23RANGE!gene68) | 1（0.02%） | 6（0.20%） | 0.07065 | 2.47E-01 | ko00670 |
| 69 | [Vascular smooth muscle contraction](../../../../C:%5CDocuments%20and%20Settings%5CChuanwang%20CAO%5C%E6%A1%8C%E9%9D%A2%5C%E6%91%87%E8%9A%8A%E8%BD%AC%E5%BD%95%E6%9C%AC%E6%95%B0%E6%8D%AE%E7%BB%93%E6%9E%9C%E6%95%B4%E7%90%860225%5CCK-vs-PT.upregulation%20invovled%20in%20pathway.xls#RANGE!gene69%23RANGE!gene69) | 86（1.69%） | 33（1.08%） | 0.071797 | 2.48E-01 | ko04270 |
| 70 | [Tuberculosis](../../../../C:%5CDocuments%20and%20Settings%5CChuanwang%20CAO%5C%E6%A1%8C%E9%9D%A2%5C%E6%91%87%E8%9A%8A%E8%BD%AC%E5%BD%95%E6%9C%AC%E6%95%B0%E6%8D%AE%E7%BB%93%E6%9E%9C%E6%95%B4%E7%90%860225%5CCK-vs-PT.upregulation%20invovled%20in%20pathway.xls#RANGE!gene70%23RANGE!gene70) | 34（0.67%） | 27（0.88%） | 0.079873 | 2.72E-01 | ko05152 |
| 71 | [Glycosaminoglycan biosynthesis - heparan sulfate](../../../../C:%5CDocuments%20and%20Settings%5CChuanwang%20CAO%5C%E6%A1%8C%E9%9D%A2%5C%E6%91%87%E8%9A%8A%E8%BD%AC%E5%BD%95%E6%9C%AC%E6%95%B0%E6%8D%AE%E7%BB%93%E6%9E%9C%E6%95%B4%E7%90%860225%5CCK-vs-PT.upregulation%20invovled%20in%20pathway.xls#RANGE!gene71%23RANGE!gene71) | 13（0.26%） | 0（0.00%） | 0.086135 | 2.89E-01 | ko00534 |
| 72 | [p53 signaling pathway](../../../../C:%5CDocuments%20and%20Settings%5CChuanwang%20CAO%5C%E6%A1%8C%E9%9D%A2%5C%E6%91%87%E8%9A%8A%E8%BD%AC%E5%BD%95%E6%9C%AC%E6%95%B0%E6%8D%AE%E7%BB%93%E6%9E%9C%E6%95%B4%E7%90%860225%5CCK-vs-PT.upregulation%20invovled%20in%20pathway.xls#RANGE!gene72%23RANGE!gene72) | 23（0.45%） | 1（0.03%） | 0.0928 | 3.07E-01 | ko04115 |
| 73 | [Glycosphingolipid biosynthesis - globo series](../../../../C:%5CDocuments%20and%20Settings%5CChuanwang%20CAO%5C%E6%A1%8C%E9%9D%A2%5C%E6%91%87%E8%9A%8A%E8%BD%AC%E5%BD%95%E6%9C%AC%E6%95%B0%E6%8D%AE%E7%BB%93%E6%9E%9C%E6%95%B4%E7%90%860225%5CCK-vs-PT.upregulation%20invovled%20in%20pathway.xls#RANGE!gene73%23RANGE!gene73) | 7（0.14%） | 4（0.13%） | 0.094657 | 3.09E-01 | ko00603 |
| 74 | [Mineral absorption](../../../../C:%5CDocuments%20and%20Settings%5CChuanwang%20CAO%5C%E6%A1%8C%E9%9D%A2%5C%E6%91%87%E8%9A%8A%E8%BD%AC%E5%BD%95%E6%9C%AC%E6%95%B0%E6%8D%AE%E7%BB%93%E6%9E%9C%E6%95%B4%E7%90%860225%5CCK-vs-PT.upregulation%20invovled%20in%20pathway.xls#RANGE!gene74%23RANGE!gene74) | 21（0.41%） | 2（0.07%） | 0.107953 | 3.47E-01 | ko04978 |
| 75 | [Taurine and hypotaurine metabolism](../../../../C:%5CDocuments%20and%20Settings%5CChuanwang%20CAO%5C%E6%A1%8C%E9%9D%A2%5C%E6%91%87%E8%9A%8A%E8%BD%AC%E5%BD%95%E6%9C%AC%E6%95%B0%E6%8D%AE%E7%BB%93%E6%9E%9C%E6%95%B4%E7%90%860225%5CCK-vs-PT.upregulation%20invovled%20in%20pathway.xls#RANGE!gene75%23RANGE!gene75) | 1（0.02%） | 6（0.20%） | 0.110139 | 3.50E-01 | ko00430 |
| 76 | [Cytosolic DNA-sensing pathway](../../../../C:%5CDocuments%20and%20Settings%5CChuanwang%20CAO%5C%E6%A1%8C%E9%9D%A2%5C%E6%91%87%E8%9A%8A%E8%BD%AC%E5%BD%95%E6%9C%AC%E6%95%B0%E6%8D%AE%E7%BB%93%E6%9E%9C%E6%95%B4%E7%90%860225%5CCK-vs-PT.upregulation%20invovled%20in%20pathway.xls#RANGE!gene76%23RANGE!gene76) | 25（0.49%） | 2（0.07%） | 0.121103 | 3.79E-01 | ko04623 |
| 77 | [Sphingolipid metabolism](../../../../C:%5CDocuments%20and%20Settings%5CChuanwang%20CAO%5C%E6%A1%8C%E9%9D%A2%5C%E6%91%87%E8%9A%8A%E8%BD%AC%E5%BD%95%E6%9C%AC%E6%95%B0%E6%8D%AE%E7%BB%93%E6%9E%9C%E6%95%B4%E7%90%860225%5CCK-vs-PT.upregulation%20invovled%20in%20pathway.xls#RANGE!gene77%23RANGE!gene77) | 9（0.18%） | 8（0.26%） | 0.126537 | 3.91E-01 | ko00600 |
| 78 | [Phenylalanine, tyrosine and tryptophan biosynthesis](../../../../C:%5CDocuments%20and%20Settings%5CChuanwang%20CAO%5C%E6%A1%8C%E9%9D%A2%5C%E6%91%87%E8%9A%8A%E8%BD%AC%E5%BD%95%E6%9C%AC%E6%95%B0%E6%8D%AE%E7%BB%93%E6%9E%9C%E6%95%B4%E7%90%860225%5CCK-vs-PT.upregulation%20invovled%20in%20pathway.xls#RANGE!gene78%23RANGE!gene78) | 0（0.00%） | 7（0.23%） | 0.13347 | 4.07E-01 | ko00400 |
| 79 | [Salivary secretion](../../../../C:%5CDocuments%20and%20Settings%5CChuanwang%20CAO%5C%E6%A1%8C%E9%9D%A2%5C%E6%91%87%E8%9A%8A%E8%BD%AC%E5%BD%95%E6%9C%AC%E6%95%B0%E6%8D%AE%E7%BB%93%E6%9E%9C%E6%95%B4%E7%90%860225%5CCK-vs-PT.upregulation%20invovled%20in%20pathway.xls#RANGE!gene79%23RANGE!gene79) | 30（0.59%） | 19（0.62%） | 0.164962 | 4.97E-01 | ko04970 |
| 80 | [Primary bile acid biosynthesis](../../../../C:%5CDocuments%20and%20Settings%5CChuanwang%20CAO%5C%E6%A1%8C%E9%9D%A2%5C%E6%91%87%E8%9A%8A%E8%BD%AC%E5%BD%95%E6%9C%AC%E6%95%B0%E6%8D%AE%E7%BB%93%E6%9E%9C%E6%95%B4%E7%90%860225%5CCK-vs-PT.upregulation%20invovled%20in%20pathway.xls#RANGE!gene80%23RANGE!gene80) | 2（0.04%） | 4（0.13%） | 0.167597 | 4.99E-01 | ko00120 |
| 81 | [Selenocompound metabolism](../../../../C:%5CDocuments%20and%20Settings%5CChuanwang%20CAO%5C%E6%A1%8C%E9%9D%A2%5C%E6%91%87%E8%9A%8A%E8%BD%AC%E5%BD%95%E6%9C%AC%E6%95%B0%E6%8D%AE%E7%BB%93%E6%9E%9C%E6%95%B4%E7%90%860225%5CCK-vs-PT.upregulation%20invovled%20in%20pathway.xls#RANGE!gene81%23RANGE!gene81) | 3（0.06%） | 7（0.23%） | 0.175639 | 5.10E-01 | ko00450 |
| 82 | [Fatty acid biosynthesis](../../../../C:%5CDocuments%20and%20Settings%5CChuanwang%20CAO%5C%E6%A1%8C%E9%9D%A2%5C%E6%91%87%E8%9A%8A%E8%BD%AC%E5%BD%95%E6%9C%AC%E6%95%B0%E6%8D%AE%E7%BB%93%E6%9E%9C%E6%95%B4%E7%90%860225%5CCK-vs-PT.upregulation%20invovled%20in%20pathway.xls#RANGE!gene82%23RANGE!gene82) | 20（0.39%） | 13（0.43%） | 0.175812 | 5.10E-01 | ko00061 |
| 83 | [Regulation of autophagy](../../../../C:%5CDocuments%20and%20Settings%5CChuanwang%20CAO%5C%E6%A1%8C%E9%9D%A2%5C%E6%91%87%E8%9A%8A%E8%BD%AC%E5%BD%95%E6%9C%AC%E6%95%B0%E6%8D%AE%E7%BB%93%E6%9E%9C%E6%95%B4%E7%90%860225%5CCK-vs-PT.upregulation%20invovled%20in%20pathway.xls#RANGE!gene83%23RANGE!gene83) | 6（0.12%） | 1（0.03%） | 0.186555 | 5.35E-01 | ko04140 |
| 84 | [Intestinal immune network for IgA production](../../../../C:%5CDocuments%20and%20Settings%5CChuanwang%20CAO%5C%E6%A1%8C%E9%9D%A2%5C%E6%91%87%E8%9A%8A%E8%BD%AC%E5%BD%95%E6%9C%AC%E6%95%B0%E6%8D%AE%E7%BB%93%E6%9E%9C%E6%95%B4%E7%90%860225%5CCK-vs-PT.upregulation%20invovled%20in%20pathway.xls#RANGE!gene84%23RANGE!gene84) | 1（0.02%） | 0（0.00%） | 0.191114 | 5.41E-01 | ko04672 |
| 85 | [Rheumatoid arthritis](../../../../C:%5CDocuments%20and%20Settings%5CChuanwang%20CAO%5C%E6%A1%8C%E9%9D%A2%5C%E6%91%87%E8%9A%8A%E8%BD%AC%E5%BD%95%E6%9C%AC%E6%95%B0%E6%8D%AE%E7%BB%93%E6%9E%9C%E6%95%B4%E7%90%860225%5CCK-vs-PT.upregulation%20invovled%20in%20pathway.xls#RANGE!gene85%23RANGE!gene85) | 11（0.22%） | 14（0.46%） | 0.210424 | 5.89E-01 | ko05323 |
| 86 | [Steroid biosynthesis](../../../../C:%5CDocuments%20and%20Settings%5CChuanwang%20CAO%5C%E6%A1%8C%E9%9D%A2%5C%E6%91%87%E8%9A%8A%E8%BD%AC%E5%BD%95%E6%9C%AC%E6%95%B0%E6%8D%AE%E7%BB%93%E6%9E%9C%E6%95%B4%E7%90%860225%5CCK-vs-PT.upregulation%20invovled%20in%20pathway.xls#RANGE!gene86%23RANGE!gene86) | 7（0.14%） | 4（0.13%） | 0.229657 | 5.99E-01 | ko00100 |
| 87 | [Jak-STAT signaling pathway](../../../../C:%5CDocuments%20and%20Settings%5CChuanwang%20CAO%5C%E6%A1%8C%E9%9D%A2%5C%E6%91%87%E8%9A%8A%E8%BD%AC%E5%BD%95%E6%9C%AC%E6%95%B0%E6%8D%AE%E7%BB%93%E6%9E%9C%E6%95%B4%E7%90%860225%5CCK-vs-PT.upregulation%20invovled%20in%20pathway.xls#RANGE!gene87%23RANGE!gene87) | 15（0.29%） | 6（0.20%） | 0.231529 | 5.99E-01 | ko04630 |
| 88 | [Arrhythmogenic right ventricular cardiomyopathy](../../../../C:%5CDocuments%20and%20Settings%5CChuanwang%20CAO%5C%E6%A1%8C%E9%9D%A2%5C%E6%91%87%E8%9A%8A%E8%BD%AC%E5%BD%95%E6%9C%AC%E6%95%B0%E6%8D%AE%E7%BB%93%E6%9E%9C%E6%95%B4%E7%90%860225%5CCK-vs-PT.upregulation%20invovled%20in%20pathway.xls#RANGE!gene88%23RANGE!gene88) （ARVC） | 23（0.45%） | 8（0.26%） | 0.232465 | 5.99E-01 | ko05412 |
| 89 | [Glycolysis / Gluconeogenesis](../../../../C:%5CDocuments%20and%20Settings%5CChuanwang%20CAO%5C%E6%A1%8C%E9%9D%A2%5C%E6%91%87%E8%9A%8A%E8%BD%AC%E5%BD%95%E6%9C%AC%E6%95%B0%E6%8D%AE%E7%BB%93%E6%9E%9C%E6%95%B4%E7%90%860225%5CCK-vs-PT.upregulation%20invovled%20in%20pathway.xls#RANGE!gene89%23RANGE!gene89) | 14（0.27%） | 8（0.26%） | 0.233723 | 5.99E-01 | ko00010 |
| 90 | [Ether lipid metabolism](../../../../C:%5CDocuments%20and%20Settings%5CChuanwang%20CAO%5C%E6%A1%8C%E9%9D%A2%5C%E6%91%87%E8%9A%8A%E8%BD%AC%E5%BD%95%E6%9C%AC%E6%95%B0%E6%8D%AE%E7%BB%93%E6%9E%9C%E6%95%B4%E7%90%860225%5CCK-vs-PT.upregulation%20invovled%20in%20pathway.xls#RANGE!gene90%23RANGE!gene90) | 13（0.26%） | 3（0.10%） | 0.234182 | 5.99E-01 | ko00565 |
| 91 | [Pyrimidine metabolism](../../../../C:%5CDocuments%20and%20Settings%5CChuanwang%20CAO%5C%E6%A1%8C%E9%9D%A2%5C%E6%91%87%E8%9A%8A%E8%BD%AC%E5%BD%95%E6%9C%AC%E6%95%B0%E6%8D%AE%E7%BB%93%E6%9E%9C%E6%95%B4%E7%90%860225%5CCK-vs-PT.upregulation%20invovled%20in%20pathway.xls#RANGE!gene91%23RANGE!gene91) | 56（1.10%） | 16（0.52%） | 0.235404 | 5.99E-01 | ko00240 |
| 92 | [Sulfur metabolism](../../../../C:%5CDocuments%20and%20Settings%5CChuanwang%20CAO%5C%E6%A1%8C%E9%9D%A2%5C%E6%91%87%E8%9A%8A%E8%BD%AC%E5%BD%95%E6%9C%AC%E6%95%B0%E6%8D%AE%E7%BB%93%E6%9E%9C%E6%95%B4%E7%90%860225%5CCK-vs-PT.upregulation%20invovled%20in%20pathway.xls#RANGE!gene92%23RANGE!gene92) | 5（0.10%） | 7（0.23%） | 0.236043 | 5.99E-01 | ko00920 |
| 93 | [Other glycan degradation](../../../../C:%5CDocuments%20and%20Settings%5CChuanwang%20CAO%5C%E6%A1%8C%E9%9D%A2%5C%E6%91%87%E8%9A%8A%E8%BD%AC%E5%BD%95%E6%9C%AC%E6%95%B0%E6%8D%AE%E7%BB%93%E6%9E%9C%E6%95%B4%E7%90%860225%5CCK-vs-PT.upregulation%20invovled%20in%20pathway.xls#RANGE!gene93%23RANGE!gene93) | 5（0.10%） | 7（0.23%） | 0.236043 | 5.99E-01 | ko00511 |
| 94 | [Prostate cancer](../../../../C:%5CDocuments%20and%20Settings%5CChuanwang%20CAO%5C%E6%A1%8C%E9%9D%A2%5C%E6%91%87%E8%9A%8A%E8%BD%AC%E5%BD%95%E6%9C%AC%E6%95%B0%E6%8D%AE%E7%BB%93%E6%9E%9C%E6%95%B4%E7%90%860225%5CCK-vs-PT.upregulation%20invovled%20in%20pathway.xls#RANGE!gene94%23RANGE!gene94) | 28（0.55%） | 9（0.29%） | 0.236754 | 5.99E-01 | ko05215 |
| 95 | [Lysine biosynthesis](../../../../C:%5CDocuments%20and%20Settings%5CChuanwang%20CAO%5C%E6%A1%8C%E9%9D%A2%5C%E6%91%87%E8%9A%8A%E8%BD%AC%E5%BD%95%E6%9C%AC%E6%95%B0%E6%8D%AE%E7%BB%93%E6%9E%9C%E6%95%B4%E7%90%860225%5CCK-vs-PT.upregulation%20invovled%20in%20pathway.xls#RANGE!gene95%23RANGE!gene95) | 1（0.02%） | 1（0.03%） | 0.244616 | 6.13E-01 | ko00300 |
| 96 | [Sulfur relay system](../../../../C:%5CDocuments%20and%20Settings%5CChuanwang%20CAO%5C%E6%A1%8C%E9%9D%A2%5C%E6%91%87%E8%9A%8A%E8%BD%AC%E5%BD%95%E6%9C%AC%E6%95%B0%E6%8D%AE%E7%BB%93%E6%9E%9C%E6%95%B4%E7%90%860225%5CCK-vs-PT.upregulation%20invovled%20in%20pathway.xls#RANGE!gene96%23RANGE!gene96) | 4（0.08%） | 1（0.03%） | 0.250692 | 6.22E-01 | ko04122 |
| 97 | [Collecting duct acid secretion](../../../../C:%5CDocuments%20and%20Settings%5CChuanwang%20CAO%5C%E6%A1%8C%E9%9D%A2%5C%E6%91%87%E8%9A%8A%E8%BD%AC%E5%BD%95%E6%9C%AC%E6%95%B0%E6%8D%AE%E7%BB%93%E6%9E%9C%E6%95%B4%E7%90%860225%5CCK-vs-PT.upregulation%20invovled%20in%20pathway.xls#RANGE!gene97%23RANGE!gene97) | 5（0.10%） | 5（0.16%） | 0.272507 | 6.62E-01 | ko04966 |
| 98 | [Gastric acid secretion](../../../../C:%5CDocuments%20and%20Settings%5CChuanwang%20CAO%5C%E6%A1%8C%E9%9D%A2%5C%E6%91%87%E8%9A%8A%E8%BD%AC%E5%BD%95%E6%9C%AC%E6%95%B0%E6%8D%AE%E7%BB%93%E6%9E%9C%E6%95%B4%E7%90%860225%5CCK-vs-PT.upregulation%20invovled%20in%20pathway.xls#RANGE!gene98%23RANGE!gene98) | 48（0.94%） | 16（0.52%） | 0.274119 | 6.62E-01 | ko04971 |
| 99 | [Natural killer cell mediated cytotoxicity](../../../../C:%5CDocuments%20and%20Settings%5CChuanwang%20CAO%5C%E6%A1%8C%E9%9D%A2%5C%E6%91%87%E8%9A%8A%E8%BD%AC%E5%BD%95%E6%9C%AC%E6%95%B0%E6%8D%AE%E7%BB%93%E6%9E%9C%E6%95%B4%E7%90%860225%5CCK-vs-PT.upregulation%20invovled%20in%20pathway.xls#RANGE!gene99%23RANGE!gene99) | 15（0.29%） | 2（0.07%） | 0.275211 | 6.62E-01 | ko04650 |
| 100 | [Pantothenate and CoA biosynthesis](../../../../C:%5CDocuments%20and%20Settings%5CChuanwang%20CAO%5C%E6%A1%8C%E9%9D%A2%5C%E6%91%87%E8%9A%8A%E8%BD%AC%E5%BD%95%E6%9C%AC%E6%95%B0%E6%8D%AE%E7%BB%93%E6%9E%9C%E6%95%B4%E7%90%860225%5CCK-vs-PT.upregulation%20invovled%20in%20pathway.xls#RANGE!gene100%23RANGE!gene100) | 4（0.08%） | 4（0.13%） | 0.287383 | 6.84E-01 | ko00770 |
| 101 | [Aldosterone-regulated sodium reabsorption](../../../../C:%5CDocuments%20and%20Settings%5CChuanwang%20CAO%5C%E6%A1%8C%E9%9D%A2%5C%E6%91%87%E8%9A%8A%E8%BD%AC%E5%BD%95%E6%9C%AC%E6%95%B0%E6%8D%AE%E7%BB%93%E6%9E%9C%E6%95%B4%E7%90%860225%5CCK-vs-PT.upregulation%20invovled%20in%20pathway.xls#RANGE!gene101%23RANGE!gene101) | 10（0.20%） | 2（0.07%） | 0.306142 | 7.20E-01 | ko04960 |
| 102 | [Circadian rhythm - mammal](../../../../C:%5CDocuments%20and%20Settings%5CChuanwang%20CAO%5C%E6%A1%8C%E9%9D%A2%5C%E6%91%87%E8%9A%8A%E8%BD%AC%E5%BD%95%E6%9C%AC%E6%95%B0%E6%8D%AE%E7%BB%93%E6%9E%9C%E6%95%B4%E7%90%860225%5CCK-vs-PT.upregulation%20invovled%20in%20pathway.xls#RANGE!gene102%23RANGE!gene102) | 11（0.22%） | 2（0.07%） | 0.308679 | 7.20E-01 | ko04710 |
| 103 | [Small cell lung cancer](../../../../C:%5CDocuments%20and%20Settings%5CChuanwang%20CAO%5C%E6%A1%8C%E9%9D%A2%5C%E6%91%87%E8%9A%8A%E8%BD%AC%E5%BD%95%E6%9C%AC%E6%95%B0%E6%8D%AE%E7%BB%93%E6%9E%9C%E6%95%B4%E7%90%860225%5CCK-vs-PT.upregulation%20invovled%20in%20pathway.xls#RANGE!gene103%23RANGE!gene103) | 22（0.43%） | 10（0.33%） | 0.329034 | 7.60E-01 | ko05222 |
| 104 | [ABC transporters](../../../../C:%5CDocuments%20and%20Settings%5CChuanwang%20CAO%5C%E6%A1%8C%E9%9D%A2%5C%E6%91%87%E8%9A%8A%E8%BD%AC%E5%BD%95%E6%9C%AC%E6%95%B0%E6%8D%AE%E7%BB%93%E6%9E%9C%E6%95%B4%E7%90%860225%5CCK-vs-PT.upregulation%20invovled%20in%20pathway.xls#RANGE!gene104%23RANGE!gene104) | 37（0.73%） | 4（0.13%） | 0.334778 | 7.60E-01 | ko02010 |
| 105 | [Malaria](../../../../C:%5CDocuments%20and%20Settings%5CChuanwang%20CAO%5C%E6%A1%8C%E9%9D%A2%5C%E6%91%87%E8%9A%8A%E8%BD%AC%E5%BD%95%E6%9C%AC%E6%95%B0%E6%8D%AE%E7%BB%93%E6%9E%9C%E6%95%B4%E7%90%860225%5CCK-vs-PT.upregulation%20invovled%20in%20pathway.xls#RANGE!gene105%23RANGE!gene105) | 8（0.16%） | 11（0.36%） | 0.335161 | 7.60E-01 | ko05144 |
| 106 | [Arginine and proline metabolism](../../../../C:%5CDocuments%20and%20Settings%5CChuanwang%20CAO%5C%E6%A1%8C%E9%9D%A2%5C%E6%91%87%E8%9A%8A%E8%BD%AC%E5%BD%95%E6%9C%AC%E6%95%B0%E6%8D%AE%E7%BB%93%E6%9E%9C%E6%95%B4%E7%90%860225%5CCK-vs-PT.upregulation%20invovled%20in%20pathway.xls#RANGE!gene106%23RANGE!gene106) | 15（0.29%） | 10（0.33%） | 0.35006 | 7.78E-01 | ko00330 |
| 107 | [Systemic lupus erythematosus](../../../../C:%5CDocuments%20and%20Settings%5CChuanwang%20CAO%5C%E6%A1%8C%E9%9D%A2%5C%E6%91%87%E8%9A%8A%E8%BD%AC%E5%BD%95%E6%9C%AC%E6%95%B0%E6%8D%AE%E7%BB%93%E6%9E%9C%E6%95%B4%E7%90%860225%5CCK-vs-PT.upregulation%20invovled%20in%20pathway.xls#RANGE!gene107%23RANGE!gene107) | 8（0.16%） | 12（0.39%） | 0.353913 | 7.78E-01 | ko05322 |
| 108 | [Glycosaminoglycan degradation](../../../../C:%5CDocuments%20and%20Settings%5CChuanwang%20CAO%5C%E6%A1%8C%E9%9D%A2%5C%E6%91%87%E8%9A%8A%E8%BD%AC%E5%BD%95%E6%9C%AC%E6%95%B0%E6%8D%AE%E7%BB%93%E6%9E%9C%E6%95%B4%E7%90%860225%5CCK-vs-PT.upregulation%20invovled%20in%20pathway.xls#RANGE!gene108%23RANGE!gene108) | 5（0.10%） | 7（0.23%） | 0.355555 | 7.78E-01 | ko00531 |
| 109 | [MAPK signaling pathway - fly](../../../../C:%5CDocuments%20and%20Settings%5CChuanwang%20CAO%5C%E6%A1%8C%E9%9D%A2%5C%E6%91%87%E8%9A%8A%E8%BD%AC%E5%BD%95%E6%9C%AC%E6%95%B0%E6%8D%AE%E7%BB%93%E6%9E%9C%E6%95%B4%E7%90%860225%5CCK-vs-PT.upregulation%20invovled%20in%20pathway.xls#RANGE!gene109%23RANGE!gene109) | 14（0.27%） | 1（0.03%） | 0.356089 | 7.78E-01 | ko04013 |
| 110 | [Hypertrophic cardiomyopathy](../../../../C:%5CDocuments%20and%20Settings%5CChuanwang%20CAO%5C%E6%A1%8C%E9%9D%A2%5C%E6%91%87%E8%9A%8A%E8%BD%AC%E5%BD%95%E6%9C%AC%E6%95%B0%E6%8D%AE%E7%BB%93%E6%9E%9C%E6%95%B4%E7%90%860225%5CCK-vs-PT.upregulation%20invovled%20in%20pathway.xls#RANGE!gene110%23RANGE!gene110) （HCM） | 47（0.92%） | 24（0.79%） | 0.404146 | 8.69E-01 | ko05410 |
| 111 | [MAPK signaling pathway](../../../../C:%5CDocuments%20and%20Settings%5CChuanwang%20CAO%5C%E6%A1%8C%E9%9D%A2%5C%E6%91%87%E8%9A%8A%E8%BD%AC%E5%BD%95%E6%9C%AC%E6%95%B0%E6%8D%AE%E7%BB%93%E6%9E%9C%E6%95%B4%E7%90%860225%5CCK-vs-PT.upregulation%20invovled%20in%20pathway.xls#RANGE!gene111%23RANGE!gene111) | 62（1.22%） | 20（0.65%） | 0.405492 | 8.69E-01 | ko04010 |
| 112 | [D-Arginine and D-ornithine metabolism](../../../../C:%5CDocuments%20and%20Settings%5CChuanwang%20CAO%5C%E6%A1%8C%E9%9D%A2%5C%E6%91%87%E8%9A%8A%E8%BD%AC%E5%BD%95%E6%9C%AC%E6%95%B0%E6%8D%AE%E7%BB%93%E6%9E%9C%E6%95%B4%E7%90%860225%5CCK-vs-PT.upregulation%20invovled%20in%20pathway.xls#RANGE!gene112%23RANGE!gene112) | 3（0.06%） | 0（0.00%） | 0.41003 | 8.71E-01 | ko00472 |
| 113 | [Dilated cardiomyopathy](../../../../C:%5CDocuments%20and%20Settings%5CChuanwang%20CAO%5C%E6%A1%8C%E9%9D%A2%5C%E6%91%87%E8%9A%8A%E8%BD%AC%E5%BD%95%E6%9C%AC%E6%95%B0%E6%8D%AE%E7%BB%93%E6%9E%9C%E6%95%B4%E7%90%860225%5CCK-vs-PT.upregulation%20invovled%20in%20pathway.xls#RANGE!gene113%23RANGE!gene113) | 48（0.94%） | 24（0.79%） | 0.422573 | 8.90E-01 | ko05414 |
| 114 | [Non-homologous end-joining](../../../../C:%5CDocuments%20and%20Settings%5CChuanwang%20CAO%5C%E6%A1%8C%E9%9D%A2%5C%E6%91%87%E8%9A%8A%E8%BD%AC%E5%BD%95%E6%9C%AC%E6%95%B0%E6%8D%AE%E7%BB%93%E6%9E%9C%E6%95%B4%E7%90%860225%5CCK-vs-PT.upregulation%20invovled%20in%20pathway.xls#RANGE!gene114%23RANGE!gene114) | 9（0.18%） | 0（0.00%） | 0.440728 | 9.20E-01 | ko03450 |
| 115 | Purine metabolism | 78（1.53%） | 21（0.69%） | 0.457426 | 9.45E-01 | ko00230 |
| 116 | [Gap junction](../../../../C:%5CDocuments%20and%20Settings%5CChuanwang%20CAO%5C%E6%A1%8C%E9%9D%A2%5C%E6%91%87%E8%9A%8A%E8%BD%AC%E5%BD%95%E6%9C%AC%E6%95%B0%E6%8D%AE%E7%BB%93%E6%9E%9C%E6%95%B4%E7%90%860225%5CCK-vs-PT.upregulation%20invovled%20in%20pathway.xls#RANGE!gene116%23RANGE!gene116) | 25（0.49%） | 3（0.10%） | 0.460512 | 9.45E-01 | ko04540 |
| 117 | [Cytokine-cytokine receptor interaction](../../../../C:%5CDocuments%20and%20Settings%5CChuanwang%20CAO%5C%E6%A1%8C%E9%9D%A2%5C%E6%91%87%E8%9A%8A%E8%BD%AC%E5%BD%95%E6%9C%AC%E6%95%B0%E6%8D%AE%E7%BB%93%E6%9E%9C%E6%95%B4%E7%90%860225%5CCK-vs-PT.upregulation%20invovled%20in%20pathway.xls#RANGE!gene117%23RANGE!gene117) | 3（0.06%） | 6（0.20%） | 0.470213 | 9.57E-01 | ko04060 |
| 118 | [Adherens junction](../../../../C:%5CDocuments%20and%20Settings%5CChuanwang%20CAO%5C%E6%A1%8C%E9%9D%A2%5C%E6%91%87%E8%9A%8A%E8%BD%AC%E5%BD%95%E6%9C%AC%E6%95%B0%E6%8D%AE%E7%BB%93%E6%9E%9C%E6%95%B4%E7%90%860225%5CCK-vs-PT.upregulation%20invovled%20in%20pathway.xls#RANGE!gene118%23RANGE!gene118) | 28（0.55%） | 12（0.39%） | 0.483555 | 9.75E-01 | ko04520 |
| 119 | [Measles](../../../../C:%5CDocuments%20and%20Settings%5CChuanwang%20CAO%5C%E6%A1%8C%E9%9D%A2%5C%E6%91%87%E8%9A%8A%E8%BD%AC%E5%BD%95%E6%9C%AC%E6%95%B0%E6%8D%AE%E7%BB%93%E6%9E%9C%E6%95%B4%E7%90%860225%5CCK-vs-PT.upregulation%20invovled%20in%20pathway.xls#RANGE!gene119%23RANGE!gene119) | 18（0.35%） | 9（0.29%） | 0.496791 | 9.94E-01 | ko05162 |
| 120 | [Biosynthesis of unsaturated fatty acids](../../../../C:%5CDocuments%20and%20Settings%5CChuanwang%20CAO%5C%E6%A1%8C%E9%9D%A2%5C%E6%91%87%E8%9A%8A%E8%BD%AC%E5%BD%95%E6%9C%AC%E6%95%B0%E6%8D%AE%E7%BB%93%E6%9E%9C%E6%95%B4%E7%90%860225%5CCK-vs-PT.upregulation%20invovled%20in%20pathway.xls#RANGE!gene120%23RANGE!gene120) | 7（0.14%） | 10（0.33%） | 0.502613 | 9.97E-01 | ko01040 |
| 121 | [Cholinergic synapse](../../../../C:%5CDocuments%20and%20Settings%5CChuanwang%20CAO%5C%E6%A1%8C%E9%9D%A2%5C%E6%91%87%E8%9A%8A%E8%BD%AC%E5%BD%95%E6%9C%AC%E6%95%B0%E6%8D%AE%E7%BB%93%E6%9E%9C%E6%95%B4%E7%90%860225%5CCK-vs-PT.upregulation%20invovled%20in%20pathway.xls#RANGE!gene121%23RANGE!gene121) | 21（0.41%） | 9（0.29%） | 0.517307 | 1.00E+00 | ko04725 |
| 122 | [NOD-like receptor signaling pathway](../../../../C:%5CDocuments%20and%20Settings%5CChuanwang%20CAO%5C%E6%A1%8C%E9%9D%A2%5C%E6%91%87%E8%9A%8A%E8%BD%AC%E5%BD%95%E6%9C%AC%E6%95%B0%E6%8D%AE%E7%BB%93%E6%9E%9C%E6%95%B4%E7%90%860225%5CCK-vs-PT.upregulation%20invovled%20in%20pathway.xls#RANGE!gene122%23RANGE!gene122) | 11（0.22%） | 2（0.07%） | 0.525008 | 1.00E+00 | ko04621 |
| 123 | [RIG-I-like receptor signaling pathway](../../../../C:%5CDocuments%20and%20Settings%5CChuanwang%20CAO%5C%E6%A1%8C%E9%9D%A2%5C%E6%91%87%E8%9A%8A%E8%BD%AC%E5%BD%95%E6%9C%AC%E6%95%B0%E6%8D%AE%E7%BB%93%E6%9E%9C%E6%95%B4%E7%90%860225%5CCK-vs-PT.upregulation%20invovled%20in%20pathway.xls#RANGE!gene123%23RANGE!gene123) | 7（0.14%） | 2（0.07%） | 0.528044 | 1.00E+00 | ko04622 |
| 124 | [Tryptophan metabolism](../../../../C:%5CDocuments%20and%20Settings%5CChuanwang%20CAO%5C%E6%A1%8C%E9%9D%A2%5C%E6%91%87%E8%9A%8A%E8%BD%AC%E5%BD%95%E6%9C%AC%E6%95%B0%E6%8D%AE%E7%BB%93%E6%9E%9C%E6%95%B4%E7%90%860225%5CCK-vs-PT.upregulation%20invovled%20in%20pathway.xls#RANGE!gene124%23RANGE!gene124) | 11（0.22%） | 4（0.13%） | 0.535005 | 1.00E+00 | ko00380 |
| 125 | [Glycosphingolipid biosynthesis - ganglio series](../../../../C:%5CDocuments%20and%20Settings%5CChuanwang%20CAO%5C%E6%A1%8C%E9%9D%A2%5C%E6%91%87%E8%9A%8A%E8%BD%AC%E5%BD%95%E6%9C%AC%E6%95%B0%E6%8D%AE%E7%BB%93%E6%9E%9C%E6%95%B4%E7%90%860225%5CCK-vs-PT.upregulation%20invovled%20in%20pathway.xls#RANGE!gene125%23RANGE!gene125) | 2（0.04%） | 4（0.13%） | 0.558364 | 1.00E+00 | ko00604 |
| 126 | [Fatty acid metabolism](../../../../C:%5CDocuments%20and%20Settings%5CChuanwang%20CAO%5C%E6%A1%8C%E9%9D%A2%5C%E6%91%87%E8%9A%8A%E8%BD%AC%E5%BD%95%E6%9C%AC%E6%95%B0%E6%8D%AE%E7%BB%93%E6%9E%9C%E6%95%B4%E7%90%860225%5CCK-vs-PT.upregulation%20invovled%20in%20pathway.xls#RANGE!gene126%23RANGE!gene126) | 12（0.24%） | 11（0.36%） | 0.565722 | 1.00E+00 | ko00071 |
| 127 | [Melanoma](../../../../C:%5CDocuments%20and%20Settings%5CChuanwang%20CAO%5C%E6%A1%8C%E9%9D%A2%5C%E6%91%87%E8%9A%8A%E8%BD%AC%E5%BD%95%E6%9C%AC%E6%95%B0%E6%8D%AE%E7%BB%93%E6%9E%9C%E6%95%B4%E7%90%860225%5CCK-vs-PT.upregulation%20invovled%20in%20pathway.xls#RANGE!gene127%23RANGE!gene127) | 8（0.16%） | 0（0.00%） | 0.566297 | 1.00E+00 | ko05218 |
| 128 | [Primary immunodeficiency](../../../../C:%5CDocuments%20and%20Settings%5CChuanwang%20CAO%5C%E6%A1%8C%E9%9D%A2%5C%E6%91%87%E8%9A%8A%E8%BD%AC%E5%BD%95%E6%9C%AC%E6%95%B0%E6%8D%AE%E7%BB%93%E6%9E%9C%E6%95%B4%E7%90%860225%5CCK-vs-PT.upregulation%20invovled%20in%20pathway.xls#RANGE!gene128%23RANGE!gene128) | 3（0.06%） | 0（0.00%） | 0.567988 | 1.00E+00 | ko05340 |
| 129 | [Adipocytokine signaling pathway](../../../../C:%5CDocuments%20and%20Settings%5CChuanwang%20CAO%5C%E6%A1%8C%E9%9D%A2%5C%E6%91%87%E8%9A%8A%E8%BD%AC%E5%BD%95%E6%9C%AC%E6%95%B0%E6%8D%AE%E7%BB%93%E6%9E%9C%E6%95%B4%E7%90%860225%5CCK-vs-PT.upregulation%20invovled%20in%20pathway.xls#RANGE!gene129%23RANGE!gene129) | 19（0.37%） | 6（0.20%） | 0.571952 | 1.00E+00 | ko04920 |
| 130 | [Renal cell carcinoma](../../../../C:%5CDocuments%20and%20Settings%5CChuanwang%20CAO%5C%E6%A1%8C%E9%9D%A2%5C%E6%91%87%E8%9A%8A%E8%BD%AC%E5%BD%95%E6%9C%AC%E6%95%B0%E6%8D%AE%E7%BB%93%E6%9E%9C%E6%95%B4%E7%90%860225%5CCK-vs-PT.upregulation%20invovled%20in%20pathway.xls#RANGE!gene130%23RANGE!gene130) | 24（0.47%） | 3（0.10%） | 0.577793 | 1.00E+00 | ko05211 |
| 131 | [T cell receptor signaling pathway](../../../../C:%5CDocuments%20and%20Settings%5CChuanwang%20CAO%5C%E6%A1%8C%E9%9D%A2%5C%E6%91%87%E8%9A%8A%E8%BD%AC%E5%BD%95%E6%9C%AC%E6%95%B0%E6%8D%AE%E7%BB%93%E6%9E%9C%E6%95%B4%E7%90%860225%5CCK-vs-PT.upregulation%20invovled%20in%20pathway.xls#RANGE!gene131%23RANGE!gene131) | 19（0.37%） | 2（0.07%） | 0.594759 | 1.00E+00 | ko04660 |
| 132 | [Glycosaminoglycan biosynthesis - keratan sulfate](../../../../C:%5CDocuments%20and%20Settings%5CChuanwang%20CAO%5C%E6%A1%8C%E9%9D%A2%5C%E6%91%87%E8%9A%8A%E8%BD%AC%E5%BD%95%E6%9C%AC%E6%95%B0%E6%8D%AE%E7%BB%93%E6%9E%9C%E6%95%B4%E7%90%860225%5CCK-vs-PT.upregulation%20invovled%20in%20pathway.xls#RANGE!gene132%23RANGE!gene132) | 2（0.04%） | 0（0.00%） | 0.596826 | 1.00E+00 | ko00533 |
| 133 | [Leukocyte transendothelial migration](../../../../C:%5CDocuments%20and%20Settings%5CChuanwang%20CAO%5C%E6%A1%8C%E9%9D%A2%5C%E6%91%87%E8%9A%8A%E8%BD%AC%E5%BD%95%E6%9C%AC%E6%95%B0%E6%8D%AE%E7%BB%93%E6%9E%9C%E6%95%B4%E7%90%860225%5CCK-vs-PT.upregulation%20invovled%20in%20pathway.xls#RANGE!gene133%23RANGE!gene133) | 35（0.69%） | 8（0.26%） | 0.603185 | 1.00E+00 | ko04670 |
| 134 | [Caffeine metabolism](../../../../C:%5CDocuments%20and%20Settings%5CChuanwang%20CAO%5C%E6%A1%8C%E9%9D%A2%5C%E6%91%87%E8%9A%8A%E8%BD%AC%E5%BD%95%E6%9C%AC%E6%95%B0%E6%8D%AE%E7%BB%93%E6%9E%9C%E6%95%B4%E7%90%860225%5CCK-vs-PT.upregulation%20invovled%20in%20pathway.xls#RANGE!gene134%23RANGE!gene134) | 3（0.06%） | 2（0.07%） | 0.608387 | 1.00E+00 | ko00232 |
| 135 | [Mucin type O-Glycan biosynthesis](../../../../C:%5CDocuments%20and%20Settings%5CChuanwang%20CAO%5C%E6%A1%8C%E9%9D%A2%5C%E6%91%87%E8%9A%8A%E8%BD%AC%E5%BD%95%E6%9C%AC%E6%95%B0%E6%8D%AE%E7%BB%93%E6%9E%9C%E6%95%B4%E7%90%860225%5CCK-vs-PT.upregulation%20invovled%20in%20pathway.xls#RANGE!gene135%23RANGE!gene135) | 8（0.16%） | 4（0.13%） | 0.625525 | 1.00E+00 | ko00512 |
| 136 | [Circadian rhythm - fly](../../../../C:%5CDocuments%20and%20Settings%5CChuanwang%20CAO%5C%E6%A1%8C%E9%9D%A2%5C%E6%91%87%E8%9A%8A%E8%BD%AC%E5%BD%95%E6%9C%AC%E6%95%B0%E6%8D%AE%E7%BB%93%E6%9E%9C%E6%95%B4%E7%90%860225%5CCK-vs-PT.upregulation%20invovled%20in%20pathway.xls#RANGE!gene136%23RANGE!gene136) | 14（0.27%） | 4（0.13%） | 0.65125 | 1.00E+00 | ko04711 |
| 137 | [Melanogenesis](../../../../C:%5CDocuments%20and%20Settings%5CChuanwang%20CAO%5C%E6%A1%8C%E9%9D%A2%5C%E6%91%87%E8%9A%8A%E8%BD%AC%E5%BD%95%E6%9C%AC%E6%95%B0%E6%8D%AE%E7%BB%93%E6%9E%9C%E6%95%B4%E7%90%860225%5CCK-vs-PT.upregulation%20invovled%20in%20pathway.xls#RANGE!gene137%23RANGE!gene137) | 16（0.31%） | 11（0.36%） | 0.653729 | 1.00E+00 | ko04916 |
| 138 | [D-Glutamine and D-glutamate metabolism](../../../../C:%5CDocuments%20and%20Settings%5CChuanwang%20CAO%5C%E6%A1%8C%E9%9D%A2%5C%E6%91%87%E8%9A%8A%E8%BD%AC%E5%BD%95%E6%9C%AC%E6%95%B0%E6%8D%AE%E7%BB%93%E6%9E%9C%E6%95%B4%E7%90%860225%5CCK-vs-PT.upregulation%20invovled%20in%20pathway.xls#RANGE!gene138%23RANGE!gene138) | 1（0.02%） | 0（0.00%） | 0.653757 | 1.00E+00 | ko00471 |
| 139 | [Vitamin B6 metabolism](../../../../C:%5CDocuments%20and%20Settings%5CChuanwang%20CAO%5C%E6%A1%8C%E9%9D%A2%5C%E6%91%87%E8%9A%8A%E8%BD%AC%E5%BD%95%E6%9C%AC%E6%95%B0%E6%8D%AE%E7%BB%93%E6%9E%9C%E6%95%B4%E7%90%860225%5CCK-vs-PT.upregulation%20invovled%20in%20pathway.xls#RANGE!gene139%23RANGE!gene139) | 1（0.02%） | 0（0.00%） | 0.653757 | 1.00E+00 | ko00750 |
| 140 | [Butirosin and neomycin biosynthesis](../../../../C:%5CDocuments%20and%20Settings%5CChuanwang%20CAO%5C%E6%A1%8C%E9%9D%A2%5C%E6%91%87%E8%9A%8A%E8%BD%AC%E5%BD%95%E6%9C%AC%E6%95%B0%E6%8D%AE%E7%BB%93%E6%9E%9C%E6%95%B4%E7%90%860225%5CCK-vs-PT.upregulation%20invovled%20in%20pathway.xls#RANGE!gene140%23RANGE!gene140) | 0（0.00%） | 1（0.03%） | 0.653757 | 1.00E+00 | ko00524 |
| 141 | [Linoleic acid metabolism](../../../../C:%5CDocuments%20and%20Settings%5CChuanwang%20CAO%5C%E6%A1%8C%E9%9D%A2%5C%E6%91%87%E8%9A%8A%E8%BD%AC%E5%BD%95%E6%9C%AC%E6%95%B0%E6%8D%AE%E7%BB%93%E6%9E%9C%E6%95%B4%E7%90%860225%5CCK-vs-PT.upregulation%20invovled%20in%20pathway.xls#RANGE!gene141%23RANGE!gene141) | 9（0.18%） | 4（0.13%） | 0.658046 | 1.00E+00 | ko00591 |
| 142 | [Fructose and mannose metabolism](../../../../C:%5CDocuments%20and%20Settings%5CChuanwang%20CAO%5C%E6%A1%8C%E9%9D%A2%5C%E6%91%87%E8%9A%8A%E8%BD%AC%E5%BD%95%E6%9C%AC%E6%95%B0%E6%8D%AE%E7%BB%93%E6%9E%9C%E6%95%B4%E7%90%860225%5CCK-vs-PT.upregulation%20invovled%20in%20pathway.xls#RANGE!gene142%23RANGE!gene142) | 9（0.18%） | 6（0.20%） | 0.658436 | 1.00E+00 | ko00051 |
| 143 | [Insulin signaling pathway](../../../../C:%5CDocuments%20and%20Settings%5CChuanwang%20CAO%5C%E6%A1%8C%E9%9D%A2%5C%E6%91%87%E8%9A%8A%E8%BD%AC%E5%BD%95%E6%9C%AC%E6%95%B0%E6%8D%AE%E7%BB%93%E6%9E%9C%E6%95%B4%E7%90%860225%5CCK-vs-PT.upregulation%20invovled%20in%20pathway.xls#RANGE!gene143%23RANGE!gene143) | 48（0.94%） | 20（0.65%） | 0.671256 | 1.00E+00 | ko04910 |
| 144 | [Aminoacyl-tRNA biosynthesis](../../../../C:%5CDocuments%20and%20Settings%5CChuanwang%20CAO%5C%E6%A1%8C%E9%9D%A2%5C%E6%91%87%E8%9A%8A%E8%BD%AC%E5%BD%95%E6%9C%AC%E6%95%B0%E6%8D%AE%E7%BB%93%E6%9E%9C%E6%95%B4%E7%90%860225%5CCK-vs-PT.upregulation%20invovled%20in%20pathway.xls#RANGE!gene144%23RANGE!gene144) | 12（0.24%） | 12（0.39%） | 0.687348 | 1.00E+00 | ko00970 |
| 145 | [Nicotinate and nicotinamide metabolism](../../../../C:%5CDocuments%20and%20Settings%5CChuanwang%20CAO%5C%E6%A1%8C%E9%9D%A2%5C%E6%91%87%E8%9A%8A%E8%BD%AC%E5%BD%95%E6%9C%AC%E6%95%B0%E6%8D%AE%E7%BB%93%E6%9E%9C%E6%95%B4%E7%90%860225%5CCK-vs-PT.upregulation%20invovled%20in%20pathway.xls#RANGE!gene145%23RANGE!gene145) | 5（0.10%） | 2（0.07%） | 0.691411 | 1.00E+00 | ko00760 |
| 146 | [Viral myocarditis](../../../../C:%5CDocuments%20and%20Settings%5CChuanwang%20CAO%5C%E6%A1%8C%E9%9D%A2%5C%E6%91%87%E8%9A%8A%E8%BD%AC%E5%BD%95%E6%9C%AC%E6%95%B0%E6%8D%AE%E7%BB%93%E6%9E%9C%E6%95%B4%E7%90%860225%5CCK-vs-PT.upregulation%20invovled%20in%20pathway.xls#RANGE!gene146%23RANGE!gene146) | 31（0.61%） | 12（0.39%） | 0.69628 | 1.00E+00 | ko05416 |
| 147 | [Type I diabetes mellitus](../../../../C:%5CDocuments%20and%20Settings%5CChuanwang%20CAO%5C%E6%A1%8C%E9%9D%A2%5C%E6%91%87%E8%9A%8A%E8%BD%AC%E5%BD%95%E6%9C%AC%E6%95%B0%E6%8D%AE%E7%BB%93%E6%9E%9C%E6%95%B4%E7%90%860225%5CCK-vs-PT.upregulation%20invovled%20in%20pathway.xls#RANGE!gene147%23RANGE!gene147) | 2（0.04%） | 0（0.00%） | 0.69918 | 1.00E+00 | ko04940 |
| 148 | [Bladder cancer](../../../../C:%5CDocuments%20and%20Settings%5CChuanwang%20CAO%5C%E6%A1%8C%E9%9D%A2%5C%E6%91%87%E8%9A%8A%E8%BD%AC%E5%BD%95%E6%9C%AC%E6%95%B0%E6%8D%AE%E7%BB%93%E6%9E%9C%E6%95%B4%E7%90%860225%5CCK-vs-PT.upregulation%20invovled%20in%20pathway.xls#RANGE!gene148%23RANGE!gene148) | 5（0.10%） | 2（0.07%） | 0.716434 | 1.00E+00 | ko05219 |
| 149 | [B cell receptor signaling pathway](../../../../C:%5CDocuments%20and%20Settings%5CChuanwang%20CAO%5C%E6%A1%8C%E9%9D%A2%5C%E6%91%87%E8%9A%8A%E8%BD%AC%E5%BD%95%E6%9C%AC%E6%95%B0%E6%8D%AE%E7%BB%93%E6%9E%9C%E6%95%B4%E7%90%860225%5CCK-vs-PT.upregulation%20invovled%20in%20pathway.xls#RANGE!gene149%23RANGE!gene149) | 10（0.20%） | 4（0.13%） | 0.723238 | 1.00E+00 | ko04662 |
| 150 | [Colorectal cancer](../../../../C:%5CDocuments%20and%20Settings%5CChuanwang%20CAO%5C%E6%A1%8C%E9%9D%A2%5C%E6%91%87%E8%9A%8A%E8%BD%AC%E5%BD%95%E6%9C%AC%E6%95%B0%E6%8D%AE%E7%BB%93%E6%9E%9C%E6%95%B4%E7%90%860225%5CCK-vs-PT.upregulation%20invovled%20in%20pathway.xls#RANGE!gene150%23RANGE!gene150) | 10（0.20%） | 4（0.13%） | 0.723238 | 1.00E+00 | ko05210 |
| 151 | [Shigellosis](../../../../C:%5CDocuments%20and%20Settings%5CChuanwang%20CAO%5C%E6%A1%8C%E9%9D%A2%5C%E6%91%87%E8%9A%8A%E8%BD%AC%E5%BD%95%E6%9C%AC%E6%95%B0%E6%8D%AE%E7%BB%93%E6%9E%9C%E6%95%B4%E7%90%860225%5CCK-vs-PT.upregulation%20invovled%20in%20pathway.xls#RANGE!gene151%23RANGE!gene151) | 22（0.43%） | 7（0.23%） | 0.734792 | 1.00E+00 | ko05131 |
| 152 | [TGF-beta signaling pathway](../../../../C:%5CDocuments%20and%20Settings%5CChuanwang%20CAO%5C%E6%A1%8C%E9%9D%A2%5C%E6%91%87%E8%9A%8A%E8%BD%AC%E5%BD%95%E6%9C%AC%E6%95%B0%E6%8D%AE%E7%BB%93%E6%9E%9C%E6%95%B4%E7%90%860225%5CCK-vs-PT.upregulation%20invovled%20in%20pathway.xls#RANGE!gene152%23RANGE!gene152) | 17（0.33%） | 5（0.16%） | 0.743865 | 1.00E+00 | ko04350 |
| 153 | [Olfactory transduction](../../../../C:%5CDocuments%20and%20Settings%5CChuanwang%20CAO%5C%E6%A1%8C%E9%9D%A2%5C%E6%91%87%E8%9A%8A%E8%BD%AC%E5%BD%95%E6%9C%AC%E6%95%B0%E6%8D%AE%E7%BB%93%E6%9E%9C%E6%95%B4%E7%90%860225%5CCK-vs-PT.upregulation%20invovled%20in%20pathway.xls#RANGE!gene153%23RANGE!gene153) | 13（0.26%） | 2（0.07%） | 0.74623 | 1.00E+00 | ko04740 |
| 154 | [Propanoate metabolism](../../../../C:%5CDocuments%20and%20Settings%5CChuanwang%20CAO%5C%E6%A1%8C%E9%9D%A2%5C%E6%91%87%E8%9A%8A%E8%BD%AC%E5%BD%95%E6%9C%AC%E6%95%B0%E6%8D%AE%E7%BB%93%E6%9E%9C%E6%95%B4%E7%90%860225%5CCK-vs-PT.upregulation%20invovled%20in%20pathway.xls#RANGE!gene154%23RANGE!gene154) | 8（0.16%） | 3（0.10%） | 0.758447 | 1.00E+00 | ko00640 |
| 155 | [Pancreatic cancer](../../../../C:%5CDocuments%20and%20Settings%5CChuanwang%20CAO%5C%E6%A1%8C%E9%9D%A2%5C%E6%91%87%E8%9A%8A%E8%BD%AC%E5%BD%95%E6%9C%AC%E6%95%B0%E6%8D%AE%E7%BB%93%E6%9E%9C%E6%95%B4%E7%90%860225%5CCK-vs-PT.upregulation%20invovled%20in%20pathway.xls#RANGE!gene155%23RANGE!gene155) | 8（0.16%） | 3（0.10%） | 0.758447 | 1.00E+00 | ko05212 |
| 156 | [VEGF signaling pathway](../../../../C:%5CDocuments%20and%20Settings%5CChuanwang%20CAO%5C%E6%A1%8C%E9%9D%A2%5C%E6%91%87%E8%9A%8A%E8%BD%AC%E5%BD%95%E6%9C%AC%E6%95%B0%E6%8D%AE%E7%BB%93%E6%9E%9C%E6%95%B4%E7%90%860225%5CCK-vs-PT.upregulation%20invovled%20in%20pathway.xls#RANGE!gene156%23RANGE!gene156) | 15（0.29%） | 4（0.13%） | 0.767861 | 1.00E+00 | ko04370 |
| 157 | [Amyotrophic lateral sclerosis](../../../../C:%5CDocuments%20and%20Settings%5CChuanwang%20CAO%5C%E6%A1%8C%E9%9D%A2%5C%E6%91%87%E8%9A%8A%E8%BD%AC%E5%BD%95%E6%9C%AC%E6%95%B0%E6%8D%AE%E7%BB%93%E6%9E%9C%E6%95%B4%E7%90%860225%5CCK-vs-PT.upregulation%20invovled%20in%20pathway.xls#RANGE!gene157%23RANGE!gene157) （ALS） | 20（0.39%） | 2（0.07%） | 0.782275 | 1.00E+00 | ko05014 |
| 158 | [Apoptosis](../../../../C:%5CDocuments%20and%20Settings%5CChuanwang%20CAO%5C%E6%A1%8C%E9%9D%A2%5C%E6%91%87%E8%9A%8A%E8%BD%AC%E5%BD%95%E6%9C%AC%E6%95%B0%E6%8D%AE%E7%BB%93%E6%9E%9C%E6%95%B4%E7%90%860225%5CCK-vs-PT.upregulation%20invovled%20in%20pathway.xls#RANGE!gene158%23RANGE!gene158) | 10（0.20%） | 0（0.00%） | 0.789347 | 1.00E+00 | ko04210 |
| 159 | [GnRH signaling pathway](../../../../C:%5CDocuments%20and%20Settings%5CChuanwang%20CAO%5C%E6%A1%8C%E9%9D%A2%5C%E6%91%87%E8%9A%8A%E8%BD%AC%E5%BD%95%E6%9C%AC%E6%95%B0%E6%8D%AE%E7%BB%93%E6%9E%9C%E6%95%B4%E7%90%860225%5CCK-vs-PT.upregulation%20invovled%20in%20pathway.xls#RANGE!gene159%23RANGE!gene159) | 22（0.43%） | 3（0.10%） | 0.795406 | 1.00E+00 | ko04912 |
| 160 | [mTOR signaling pathway](../../../../C:%5CDocuments%20and%20Settings%5CChuanwang%20CAO%5C%E6%A1%8C%E9%9D%A2%5C%E6%91%87%E8%9A%8A%E8%BD%AC%E5%BD%95%E6%9C%AC%E6%95%B0%E6%8D%AE%E7%BB%93%E6%9E%9C%E6%95%B4%E7%90%860225%5CCK-vs-PT.upregulation%20invovled%20in%20pathway.xls#RANGE!gene160%23RANGE!gene160) | 16（0.31%） | 1（0.03%） | 0.798726 | 1.00E+00 | ko04150 |
| 161 | [beta-Alanine metabolism](../../../../C:%5CDocuments%20and%20Settings%5CChuanwang%20CAO%5C%E6%A1%8C%E9%9D%A2%5C%E6%91%87%E8%9A%8A%E8%BD%AC%E5%BD%95%E6%9C%AC%E6%95%B0%E6%8D%AE%E7%BB%93%E6%9E%9C%E6%95%B4%E7%90%860225%5CCK-vs-PT.upregulation%20invovled%20in%20pathway.xls#RANGE!gene161%23RANGE!gene161) | 3（0.06%） | 7（0.23%） | 0.805688 | 1.00E+00 | ko00410 |
| 162 | [mRNA surveillance pathway](../../../../C:%5CDocuments%20and%20Settings%5CChuanwang%20CAO%5C%E6%A1%8C%E9%9D%A2%5C%E6%91%87%E8%9A%8A%E8%BD%AC%E5%BD%95%E6%9C%AC%E6%95%B0%E6%8D%AE%E7%BB%93%E6%9E%9C%E6%95%B4%E7%90%860225%5CCK-vs-PT.upregulation%20invovled%20in%20pathway.xls#RANGE!gene162%23RANGE!gene162) | 40（0.79%） | 3（0.10%） | 0.810766 | 1.00E+00 | ko03015 |
| 163 | [Type II diabetes mellitus](../../../../C:%5CDocuments%20and%20Settings%5CChuanwang%20CAO%5C%E6%A1%8C%E9%9D%A2%5C%E6%91%87%E8%9A%8A%E8%BD%AC%E5%BD%95%E6%9C%AC%E6%95%B0%E6%8D%AE%E7%BB%93%E6%9E%9C%E6%95%B4%E7%90%860225%5CCK-vs-PT.upregulation%20invovled%20in%20pathway.xls#RANGE!gene163%23RANGE!gene163) | 9（0.18%） | 1（0.03%） | 0.821066 | 1.00E+00 | ko04930 |
| 164 | [Protein processing in endoplasmic reticulum](../../../../C:%5CDocuments%20and%20Settings%5CChuanwang%20CAO%5C%E6%A1%8C%E9%9D%A2%5C%E6%91%87%E8%9A%8A%E8%BD%AC%E5%BD%95%E6%9C%AC%E6%95%B0%E6%8D%AE%E7%BB%93%E6%9E%9C%E6%95%B4%E7%90%860225%5CCK-vs-PT.upregulation%20invovled%20in%20pathway.xls#RANGE!gene164%23RANGE!gene164) | 42（0.82%） | 7（0.23%） | 0.824269 | 1.00E+00 | ko04141 |
| 165 | [Cardiac muscle contraction](../../../../C:%5CDocuments%20and%20Settings%5CChuanwang%20CAO%5C%E6%A1%8C%E9%9D%A2%5C%E6%91%87%E8%9A%8A%E8%BD%AC%E5%BD%95%E6%9C%AC%E6%95%B0%E6%8D%AE%E7%BB%93%E6%9E%9C%E6%95%B4%E7%90%860225%5CCK-vs-PT.upregulation%20invovled%20in%20pathway.xls#RANGE!gene165%23RANGE!gene165) | 25（0.49%） | 9（0.29%） | 0.828756 | 1.00E+00 | ko04260 |
| 166 | [Fc gamma R-mediated phagocytosis](../../../../C:%5CDocuments%20and%20Settings%5CChuanwang%20CAO%5C%E6%A1%8C%E9%9D%A2%5C%E6%91%87%E8%9A%8A%E8%BD%AC%E5%BD%95%E6%9C%AC%E6%95%B0%E6%8D%AE%E7%BB%93%E6%9E%9C%E6%95%B4%E7%90%860225%5CCK-vs-PT.upregulation%20invovled%20in%20pathway.xls#RANGE!gene166%23RANGE!gene166) | 27（0.53%） | 9（0.29%） | 0.835164 | 1.00E+00 | ko04666 |
| 167 | [Maturity onset diabetes of the young](../../../../C:%5CDocuments%20and%20Settings%5CChuanwang%20CAO%5C%E6%A1%8C%E9%9D%A2%5C%E6%91%87%E8%9A%8A%E8%BD%AC%E5%BD%95%E6%9C%AC%E6%95%B0%E6%8D%AE%E7%BB%93%E6%9E%9C%E6%95%B4%E7%90%860225%5CCK-vs-PT.upregulation%20invovled%20in%20pathway.xls#RANGE!gene167%23RANGE!gene167) | 1（0.02%） | 2（0.07%） | 0.843718 | 1.00E+00 | ko04950 |
| 168 | [Dorso-ventral axis formation](../../../../C:%5CDocuments%20and%20Settings%5CChuanwang%20CAO%5C%E6%A1%8C%E9%9D%A2%5C%E6%91%87%E8%9A%8A%E8%BD%AC%E5%BD%95%E6%9C%AC%E6%95%B0%E6%8D%AE%E7%BB%93%E6%9E%9C%E6%95%B4%E7%90%860225%5CCK-vs-PT.upregulation%20invovled%20in%20pathway.xls#RANGE!gene168%23RANGE!gene168) | 30（0.59%） | 7（0.23%） | 0.845916 | 1.00E+00 | ko04320 |
| 169 | [Biotin metabolism](../../../../C:%5CDocuments%20and%20Settings%5CChuanwang%20CAO%5C%E6%A1%8C%E9%9D%A2%5C%E6%91%87%E8%9A%8A%E8%BD%AC%E5%BD%95%E6%9C%AC%E6%95%B0%E6%8D%AE%E7%BB%93%E6%9E%9C%E6%95%B4%E7%90%860225%5CCK-vs-PT.upregulation%20invovled%20in%20pathway.xls#RANGE!gene169%23RANGE!gene169) | 1（0.02%） | 0（0.00%） | 0.851822 | 1.00E+00 | ko00780 |
| 170 | [Hedgehog signaling pathway](../../../../C:%5CDocuments%20and%20Settings%5CChuanwang%20CAO%5C%E6%A1%8C%E9%9D%A2%5C%E6%91%87%E8%9A%8A%E8%BD%AC%E5%BD%95%E6%9C%AC%E6%95%B0%E6%8D%AE%E7%BB%93%E6%9E%9C%E6%95%B4%E7%90%860225%5CCK-vs-PT.upregulation%20invovled%20in%20pathway.xls#RANGE!gene170%23RANGE!gene170) | 8（0.16%） | 5（0.16%） | 0.861832 | 1.00E+00 | ko04340 |
| 171 | [Cell adhesion molecules](../../../../C:%5CDocuments%20and%20Settings%5CChuanwang%20CAO%5C%E6%A1%8C%E9%9D%A2%5C%E6%91%87%E8%9A%8A%E8%BD%AC%E5%BD%95%E6%9C%AC%E6%95%B0%E6%8D%AE%E7%BB%93%E6%9E%9C%E6%95%B4%E7%90%860225%5CCK-vs-PT.upregulation%20invovled%20in%20pathway.xls#RANGE!gene171%23RANGE!gene171) （CAMs） | 21（0.41%） | 4（0.13%） | 0.862465 | 1.00E+00 | ko04514 |
| 172 | [Neurotrophin signaling pathway](../../../../C:%5CDocuments%20and%20Settings%5CChuanwang%20CAO%5C%E6%A1%8C%E9%9D%A2%5C%E6%91%87%E8%9A%8A%E8%BD%AC%E5%BD%95%E6%9C%AC%E6%95%B0%E6%8D%AE%E7%BB%93%E6%9E%9C%E6%95%B4%E7%90%860225%5CCK-vs-PT.upregulation%20invovled%20in%20pathway.xls#RANGE!gene172%23RANGE!gene172) | 24（0.47%） | 5（0.16%） | 0.863299 | 1.00E+00 | ko04722 |
| 173 | [Alzheimer's disease](../../../../C:%5CDocuments%20and%20Settings%5CChuanwang%20CAO%5C%E6%A1%8C%E9%9D%A2%5C%E6%91%87%E8%9A%8A%E8%BD%AC%E5%BD%95%E6%9C%AC%E6%95%B0%E6%8D%AE%E7%BB%93%E6%9E%9C%E6%95%B4%E7%90%860225%5CCK-vs-PT.upregulation%20invovled%20in%20pathway.xls#RANGE!gene173%23RANGE!gene173) | 28（0.55%） | 26（0.85%） | 0.864694 | 1.00E+00 | ko05010 |
| 174 | [Pentose phosphate pathway](../../../../C:%5CDocuments%20and%20Settings%5CChuanwang%20CAO%5C%E6%A1%8C%E9%9D%A2%5C%E6%91%87%E8%9A%8A%E8%BD%AC%E5%BD%95%E6%9C%AC%E6%95%B0%E6%8D%AE%E7%BB%93%E6%9E%9C%E6%95%B4%E7%90%860225%5CCK-vs-PT.upregulation%20invovled%20in%20pathway.xls#RANGE!gene174%23RANGE!gene174) | 4（0.08%） | 6（0.20%） | 0.884369 | 1.00E+00 | ko00030 |
| 175 | [Thyroid cancer](../../../../C:%5CDocuments%20and%20Settings%5CChuanwang%20CAO%5C%E6%A1%8C%E9%9D%A2%5C%E6%91%87%E8%9A%8A%E8%BD%AC%E5%BD%95%E6%9C%AC%E6%95%B0%E6%8D%AE%E7%BB%93%E6%9E%9C%E6%95%B4%E7%90%860225%5CCK-vs-PT.upregulation%20invovled%20in%20pathway.xls#RANGE!gene175%23RANGE!gene175) | 2（0.04%） | 3（0.10%） | 0.890736 | 1.00E+00 | ko05216 |
| 176 | [RNA degradation](../../../../C:%5CDocuments%20and%20Settings%5CChuanwang%20CAO%5C%E6%A1%8C%E9%9D%A2%5C%E6%91%87%E8%9A%8A%E8%BD%AC%E5%BD%95%E6%9C%AC%E6%95%B0%E6%8D%AE%E7%BB%93%E6%9E%9C%E6%95%B4%E7%90%860225%5CCK-vs-PT.upregulation%20invovled%20in%20pathway.xls#RANGE!gene176%23RANGE!gene176) | 38（0.75%） | 11（0.36%） | 0.892082 | 1.00E+00 | ko03018 |
| 177 | [Phototransduction - fly](../../../../C:%5CDocuments%20and%20Settings%5CChuanwang%20CAO%5C%E6%A1%8C%E9%9D%A2%5C%E6%91%87%E8%9A%8A%E8%BD%AC%E5%BD%95%E6%9C%AC%E6%95%B0%E6%8D%AE%E7%BB%93%E6%9E%9C%E6%95%B4%E7%90%860225%5CCK-vs-PT.upregulation%20invovled%20in%20pathway.xls#RANGE!gene177%23RANGE!gene177) | 12（0.24%） | 6（0.20%） | 0.900236 | 1.00E+00 | ko04745 |
| 178 | [Fc epsilon RI signaling pathway](../../../../C:%5CDocuments%20and%20Settings%5CChuanwang%20CAO%5C%E6%A1%8C%E9%9D%A2%5C%E6%91%87%E8%9A%8A%E8%BD%AC%E5%BD%95%E6%9C%AC%E6%95%B0%E6%8D%AE%E7%BB%93%E6%9E%9C%E6%95%B4%E7%90%860225%5CCK-vs-PT.upregulation%20invovled%20in%20pathway.xls#RANGE!gene178%23RANGE!gene178) | 9（0.18%） | 4（0.13%） | 0.900338 | 1.00E+00 | ko04664 |
| 179 | [Glycosphingolipid biosynthesis - lacto and neolacto series](../../../../C:%5CDocuments%20and%20Settings%5CChuanwang%20CAO%5C%E6%A1%8C%E9%9D%A2%5C%E6%91%87%E8%9A%8A%E8%BD%AC%E5%BD%95%E6%9C%AC%E6%95%B0%E6%8D%AE%E7%BB%93%E6%9E%9C%E6%95%B4%E7%90%860225%5CCK-vs-PT.upregulation%20invovled%20in%20pathway.xls#RANGE!gene179%23RANGE!gene179) | 1（0.02%） | 0（0.00%） | 0.903071 | 1.00E+00 | ko00601 |
| 180 | [Bacterial invasion of epithelial cells](../../../../C:%5CDocuments%20and%20Settings%5CChuanwang%20CAO%5C%E6%A1%8C%E9%9D%A2%5C%E6%91%87%E8%9A%8A%E8%BD%AC%E5%BD%95%E6%9C%AC%E6%95%B0%E6%8D%AE%E7%BB%93%E6%9E%9C%E6%95%B4%E7%90%860225%5CCK-vs-PT.upregulation%20invovled%20in%20pathway.xls#RANGE!gene180%23RANGE!gene180) | 24（0.47%） | 8（0.26%） | 0.91265 | 1.00E+00 | ko05100 |
| 181 | [Pathways in cancer](../../../../C:%5CDocuments%20and%20Settings%5CChuanwang%20CAO%5C%E6%A1%8C%E9%9D%A2%5C%E6%91%87%E8%9A%8A%E8%BD%AC%E5%BD%95%E6%9C%AC%E6%95%B0%E6%8D%AE%E7%BB%93%E6%9E%9C%E6%95%B4%E7%90%860225%5CCK-vs-PT.upregulation%20invovled%20in%20pathway.xls#RANGE!gene181%23RANGE!gene181) | 72（1.41%） | 26（0.85%） | 0.91374 | 1.00E+00 | ko05200 |
| 182 | [Histidine metabolism](../../../../C:%5CDocuments%20and%20Settings%5CChuanwang%20CAO%5C%E6%A1%8C%E9%9D%A2%5C%E6%91%87%E8%9A%8A%E8%BD%AC%E5%BD%95%E6%9C%AC%E6%95%B0%E6%8D%AE%E7%BB%93%E6%9E%9C%E6%95%B4%E7%90%860225%5CCK-vs-PT.upregulation%20invovled%20in%20pathway.xls#RANGE!gene182%23RANGE!gene182) | 3（0.06%） | 2（0.07%） | 0.914499 | 1.00E+00 | ko00340 |
| 183 | [Glioma](../../../../C:%5CDocuments%20and%20Settings%5CChuanwang%20CAO%5C%E6%A1%8C%E9%9D%A2%5C%E6%91%87%E8%9A%8A%E8%BD%AC%E5%BD%95%E6%9C%AC%E6%95%B0%E6%8D%AE%E7%BB%93%E6%9E%9C%E6%95%B4%E7%90%860225%5CCK-vs-PT.upregulation%20invovled%20in%20pathway.xls#RANGE!gene183%23RANGE!gene183) | 12（0.24%） | 3（0.10%） | 0.914591 | 1.00E+00 | ko05214 |
| 184 | [Base excision repair](../../../../C:%5CDocuments%20and%20Settings%5CChuanwang%20CAO%5C%E6%A1%8C%E9%9D%A2%5C%E6%91%87%E8%9A%8A%E8%BD%AC%E5%BD%95%E6%9C%AC%E6%95%B0%E6%8D%AE%E7%BB%93%E6%9E%9C%E6%95%B4%E7%90%860225%5CCK-vs-PT.upregulation%20invovled%20in%20pathway.xls#RANGE!gene184%23RANGE!gene184) | 8（0.16%） | 2（0.07%） | 0.920654 | 1.00E+00 | ko03410 |
| 185 | [Endocrine and other factor-regulated calcium reabsorption](../../../../C:%5CDocuments%20and%20Settings%5CChuanwang%20CAO%5C%E6%A1%8C%E9%9D%A2%5C%E6%91%87%E8%9A%8A%E8%BD%AC%E5%BD%95%E6%9C%AC%E6%95%B0%E6%8D%AE%E7%BB%93%E6%9E%9C%E6%95%B4%E7%90%860225%5CCK-vs-PT.upregulation%20invovled%20in%20pathway.xls#RANGE!gene185%23RANGE!gene185) | 10（0.20%） | 4（0.13%） | 0.928457 | 1.00E+00 | ko04961 |
| 186 | [Valine, leucine and isoleucine degradation](../../../../C:%5CDocuments%20and%20Settings%5CChuanwang%20CAO%5C%E6%A1%8C%E9%9D%A2%5C%E6%91%87%E8%9A%8A%E8%BD%AC%E5%BD%95%E6%9C%AC%E6%95%B0%E6%8D%AE%E7%BB%93%E6%9E%9C%E6%95%B4%E7%90%860225%5CCK-vs-PT.upregulation%20invovled%20in%20pathway.xls#RANGE!gene186%23RANGE!gene186) | 7（0.14%） | 7（0.23%） | 0.928457 | 1.00E+00 | ko00280 |
| 187 | [Phototransduction](../../../../C:%5CDocuments%20and%20Settings%5CChuanwang%20CAO%5C%E6%A1%8C%E9%9D%A2%5C%E6%91%87%E8%9A%8A%E8%BD%AC%E5%BD%95%E6%9C%AC%E6%95%B0%E6%8D%AE%E7%BB%93%E6%9E%9C%E6%95%B4%E7%90%860225%5CCK-vs-PT.upregulation%20invovled%20in%20pathway.xls#RANGE!gene187%23RANGE!gene187) | 6（0.12%） | 1（0.03%） | 0.930354 | 1.00E+00 | ko04744 |
| 188 | [Alanine, aspartate and glutamate metabolism](../../../../C:%5CDocuments%20and%20Settings%5CChuanwang%20CAO%5C%E6%A1%8C%E9%9D%A2%5C%E6%91%87%E8%9A%8A%E8%BD%AC%E5%BD%95%E6%9C%AC%E6%95%B0%E6%8D%AE%E7%BB%93%E6%9E%9C%E6%95%B4%E7%90%860225%5CCK-vs-PT.upregulation%20invovled%20in%20pathway.xls#RANGE!gene188%23RANGE!gene188) | 4（0.08%） | 5（0.16%） | 0.930637 | 1.00E+00 | ko00250 |
| 189 | [Acute myeloid leukemia](../../../../C:%5CDocuments%20and%20Settings%5CChuanwang%20CAO%5C%E6%A1%8C%E9%9D%A2%5C%E6%91%87%E8%9A%8A%E8%BD%AC%E5%BD%95%E6%9C%AC%E6%95%B0%E6%8D%AE%E7%BB%93%E6%9E%9C%E6%95%B4%E7%90%860225%5CCK-vs-PT.upregulation%20invovled%20in%20pathway.xls#RANGE!gene189%23RANGE!gene189) | 6（0.12%） | 0（0.00%） | 0.93536 | 1.00E+00 | ko05221 |
| 190 | [Fatty acid elongation](../../../../C:%5CDocuments%20and%20Settings%5CChuanwang%20CAO%5C%E6%A1%8C%E9%9D%A2%5C%E6%91%87%E8%9A%8A%E8%BD%AC%E5%BD%95%E6%9C%AC%E6%95%B0%E6%8D%AE%E7%BB%93%E6%9E%9C%E6%95%B4%E7%90%860225%5CCK-vs-PT.upregulation%20invovled%20in%20pathway.xls#RANGE!gene190%23RANGE!gene190) | 2（0.04%） | 0（0.00%） | 0.941791 | 1.00E+00 | ko00062 |
| 191 | [Prion diseases](../../../../C:%5CDocuments%20and%20Settings%5CChuanwang%20CAO%5C%E6%A1%8C%E9%9D%A2%5C%E6%91%87%E8%9A%8A%E8%BD%AC%E5%BD%95%E6%9C%AC%E6%95%B0%E6%8D%AE%E7%BB%93%E6%9E%9C%E6%95%B4%E7%90%860225%5CCK-vs-PT.upregulation%20invovled%20in%20pathway.xls#RANGE!gene191%23RANGE!gene191) | 12（0.24%） | 3（0.10%） | 0.943997 | 1.00E+00 | ko05020 |
| 192 | [Calcium signaling pathway](../../../../C:%5CDocuments%20and%20Settings%5CChuanwang%20CAO%5C%E6%A1%8C%E9%9D%A2%5C%E6%91%87%E8%9A%8A%E8%BD%AC%E5%BD%95%E6%9C%AC%E6%95%B0%E6%8D%AE%E7%BB%93%E6%9E%9C%E6%95%B4%E7%90%860225%5CCK-vs-PT.upregulation%20invovled%20in%20pathway.xls#RANGE!gene192%23RANGE!gene192) | 48（0.94%） | 10（0.33%） | 0.950416 | 1.00E+00 | ko04020 |
| 193 | [Chagas disease](../../../../C:%5CDocuments%20and%20Settings%5CChuanwang%20CAO%5C%E6%A1%8C%E9%9D%A2%5C%E6%91%87%E8%9A%8A%E8%BD%AC%E5%BD%95%E6%9C%AC%E6%95%B0%E6%8D%AE%E7%BB%93%E6%9E%9C%E6%95%B4%E7%90%860225%5CCK-vs-PT.upregulation%20invovled%20in%20pathway.xls#RANGE!gene193%23RANGE!gene193) （American trypanosomiasis） | 7（0.14%） | 6（0.20%） | 0.955525 | 1.00E+00 | ko05142 |
| 194 | [Citrate cycle](../../../../C:%5CDocuments%20and%20Settings%5CChuanwang%20CAO%5C%E6%A1%8C%E9%9D%A2%5C%E6%91%87%E8%9A%8A%E8%BD%AC%E5%BD%95%E6%9C%AC%E6%95%B0%E6%8D%AE%E7%BB%93%E6%9E%9C%E6%95%B4%E7%90%860225%5CCK-vs-PT.upregulation%20invovled%20in%20pathway.xls#RANGE!gene194%23RANGE!gene194) （TCA cycle） | 9（0.18%） | 0（0.00%） | 0.959235 | 1.00E+00 | ko00020 |
| 195 | [SNARE interactions in vesicular transport](../../../../C:%5CDocuments%20and%20Settings%5CChuanwang%20CAO%5C%E6%A1%8C%E9%9D%A2%5C%E6%91%87%E8%9A%8A%E8%BD%AC%E5%BD%95%E6%9C%AC%E6%95%B0%E6%8D%AE%E7%BB%93%E6%9E%9C%E6%95%B4%E7%90%860225%5CCK-vs-PT.upregulation%20invovled%20in%20pathway.xls#RANGE!gene195%23RANGE!gene195) | 5（0.10%） | 0（0.00%） | 0.960728 | 1.00E+00 | ko04130 |
| 196 | [Taste transduction](../../../../C:%5CDocuments%20and%20Settings%5CChuanwang%20CAO%5C%E6%A1%8C%E9%9D%A2%5C%E6%91%87%E8%9A%8A%E8%BD%AC%E5%BD%95%E6%9C%AC%E6%95%B0%E6%8D%AE%E7%BB%93%E6%9E%9C%E6%95%B4%E7%90%860225%5CCK-vs-PT.upregulation%20invovled%20in%20pathway.xls#RANGE!gene196%23RANGE!gene196) | 4（0.08%） | 1（0.03%） | 0.960728 | 1.00E+00 | ko04742 |
| 197 | [Endocytosis](../../../../C:%5CDocuments%20and%20Settings%5CChuanwang%20CAO%5C%E6%A1%8C%E9%9D%A2%5C%E6%91%87%E8%9A%8A%E8%BD%AC%E5%BD%95%E6%9C%AC%E6%95%B0%E6%8D%AE%E7%BB%93%E6%9E%9C%E6%95%B4%E7%90%860225%5CCK-vs-PT.upregulation%20invovled%20in%20pathway.xls#RANGE!gene197%23RANGE!gene197) | 43（0.84%） | 13（0.43%） | 0.962864 | 1.00E+00 | ko04144 |
| 198 | [Progesterone-mediated oocyte maturation](../../../../C:%5CDocuments%20and%20Settings%5CChuanwang%20CAO%5C%E6%A1%8C%E9%9D%A2%5C%E6%91%87%E8%9A%8A%E8%BD%AC%E5%BD%95%E6%9C%AC%E6%95%B0%E6%8D%AE%E7%BB%93%E6%9E%9C%E6%95%B4%E7%90%860225%5CCK-vs-PT.upregulation%20invovled%20in%20pathway.xls#RANGE!gene198%23RANGE!gene198) | 18（0.35%） | 4（0.13%） | 0.96556 | 1.00E+00 | ko04914 |
| 199 | [Synthesis and degradation of ketone bodies](../../../../C:%5CDocuments%20and%20Settings%5CChuanwang%20CAO%5C%E6%A1%8C%E9%9D%A2%5C%E6%91%87%E8%9A%8A%E8%BD%AC%E5%BD%95%E6%9C%AC%E6%95%B0%E6%8D%AE%E7%BB%93%E6%9E%9C%E6%95%B4%E7%90%860225%5CCK-vs-PT.upregulation%20invovled%20in%20pathway.xls#RANGE!gene199%23RANGE!gene199) | 0（0.00%） | 1（0.03%） | 0.966463 | 1.00E+00 | ko00072 |
| 200 | [ErbB signaling pathway](../../../../C:%5CDocuments%20and%20Settings%5CChuanwang%20CAO%5C%E6%A1%8C%E9%9D%A2%5C%E6%91%87%E8%9A%8A%E8%BD%AC%E5%BD%95%E6%9C%AC%E6%95%B0%E6%8D%AE%E7%BB%93%E6%9E%9C%E6%95%B4%E7%90%860225%5CCK-vs-PT.upregulation%20invovled%20in%20pathway.xls#RANGE!gene200%23RANGE!gene200) | 13（0.26%） | 3（0.10%） | 0.969324 | 1.00E+00 | ko04012 |
| 201 | [Proteasome](../../../../C:%5CDocuments%20and%20Settings%5CChuanwang%20CAO%5C%E6%A1%8C%E9%9D%A2%5C%E6%91%87%E8%9A%8A%E8%BD%AC%E5%BD%95%E6%9C%AC%E6%95%B0%E6%8D%AE%E7%BB%93%E6%9E%9C%E6%95%B4%E7%90%860225%5CCK-vs-PT.upregulation%20invovled%20in%20pathway.xls#RANGE!gene201%23RANGE!gene201) | 5（0.10%） | 1（0.03%） | 0.969468 | 1.00E+00 | ko03050 |
| 202 | [Wnt signaling pathway](../../../../C:%5CDocuments%20and%20Settings%5CChuanwang%20CAO%5C%E6%A1%8C%E9%9D%A2%5C%E6%91%87%E8%9A%8A%E8%BD%AC%E5%BD%95%E6%9C%AC%E6%95%B0%E6%8D%AE%E7%BB%93%E6%9E%9C%E6%95%B4%E7%90%860225%5CCK-vs-PT.upregulation%20invovled%20in%20pathway.xls#RANGE!gene202%23RANGE!gene202) | 30（0.59%） | 7（0.23%） | 0.971247 | 1.00E+00 | ko04310 |
| 203 | [Epithelial cell signaling in Helicobacter pylori infection](../../../../C:%5CDocuments%20and%20Settings%5CChuanwang%20CAO%5C%E6%A1%8C%E9%9D%A2%5C%E6%91%87%E8%9A%8A%E8%BD%AC%E5%BD%95%E6%9C%AC%E6%95%B0%E6%8D%AE%E7%BB%93%E6%9E%9C%E6%95%B4%E7%90%860225%5CCK-vs-PT.upregulation%20invovled%20in%20pathway.xls#RANGE!gene203%23RANGE!gene203) | 8（0.16%） | 3（0.10%） | 0.972911 | 1.00E+00 | ko05120 |
| 204 | [Endometrial cancer](../../../../C:%5CDocuments%20and%20Settings%5CChuanwang%20CAO%5C%E6%A1%8C%E9%9D%A2%5C%E6%91%87%E8%9A%8A%E8%BD%AC%E5%BD%95%E6%9C%AC%E6%95%B0%E6%8D%AE%E7%BB%93%E6%9E%9C%E6%95%B4%E7%90%860225%5CCK-vs-PT.upregulation%20invovled%20in%20pathway.xls#RANGE!gene204%23RANGE!gene204) | 5（0.10%） | 2（0.07%） | 0.973066 | 1.00E+00 | ko05213 |
| 205 | [Glutamatergic synapse](../../../../C:%5CDocuments%20and%20Settings%5CChuanwang%20CAO%5C%E6%A1%8C%E9%9D%A2%5C%E6%91%87%E8%9A%8A%E8%BD%AC%E5%BD%95%E6%9C%AC%E6%95%B0%E6%8D%AE%E7%BB%93%E6%9E%9C%E6%95%B4%E7%90%860225%5CCK-vs-PT.upregulation%20invovled%20in%20pathway.xls#RANGE!gene205%23RANGE!gene205) | 22（0.43%） | 4（0.13%） | 0.973825 | 1.00E+00 | ko04724 |
| 206 | [Terpenoid backbone biosynthesis](../../../../C:%5CDocuments%20and%20Settings%5CChuanwang%20CAO%5C%E6%A1%8C%E9%9D%A2%5C%E6%91%87%E8%9A%8A%E8%BD%AC%E5%BD%95%E6%9C%AC%E6%95%B0%E6%8D%AE%E7%BB%93%E6%9E%9C%E6%95%B4%E7%90%860225%5CCK-vs-PT.upregulation%20invovled%20in%20pathway.xls#RANGE!gene206%23RANGE!gene206) | 1（0.02%） | 2（0.07%） | 0.974731 | 1.00E+00 | ko00900 |
| 207 | [Basal cell carcinoma](../../../../C:%5CDocuments%20and%20Settings%5CChuanwang%20CAO%5C%E6%A1%8C%E9%9D%A2%5C%E6%91%87%E8%9A%8A%E8%BD%AC%E5%BD%95%E6%9C%AC%E6%95%B0%E6%8D%AE%E7%BB%93%E6%9E%9C%E6%95%B4%E7%90%860225%5CCK-vs-PT.upregulation%20invovled%20in%20pathway.xls#RANGE!gene207%23RANGE!gene207) | 4（0.08%） | 2（0.07%） | 0.976487 | 1.00E+00 | ko05217 |
| 208 | [Chemokine signaling pathway](../../../../C:%5CDocuments%20and%20Settings%5CChuanwang%20CAO%5C%E6%A1%8C%E9%9D%A2%5C%E6%91%87%E8%9A%8A%E8%BD%AC%E5%BD%95%E6%9C%AC%E6%95%B0%E6%8D%AE%E7%BB%93%E6%9E%9C%E6%95%B4%E7%90%860225%5CCK-vs-PT.upregulation%20invovled%20in%20pathway.xls#RANGE!gene208%23RANGE!gene208) | 25（0.49%） | 7（0.23%） | 0.976631 | 1.00E+00 | ko04062 |
| 209 | [Butanoate metabolism](../../../../C:%5CDocuments%20and%20Settings%5CChuanwang%20CAO%5C%E6%A1%8C%E9%9D%A2%5C%E6%91%87%E8%9A%8A%E8%BD%AC%E5%BD%95%E6%9C%AC%E6%95%B0%E6%8D%AE%E7%BB%93%E6%9E%9C%E6%95%B4%E7%90%860225%5CCK-vs-PT.upregulation%20invovled%20in%20pathway.xls#RANGE!gene209%23RANGE!gene209) | 7（0.14%） | 2（0.07%） | 0.979333 | 1.00E+00 | ko00650 |
| 210 | [Lysine degradation](../../../../C:%5CDocuments%20and%20Settings%5CChuanwang%20CAO%5C%E6%A1%8C%E9%9D%A2%5C%E6%91%87%E8%9A%8A%E8%BD%AC%E5%BD%95%E6%9C%AC%E6%95%B0%E6%8D%AE%E7%BB%93%E6%9E%9C%E6%95%B4%E7%90%860225%5CCK-vs-PT.upregulation%20invovled%20in%20pathway.xls#RANGE!gene210%23RANGE!gene210) | 20（0.39%） | 10（0.33%） | 0.980058 | 1.00E+00 | ko00310 |
| 211 | [Notch signaling pathway](../../../../C:%5CDocuments%20and%20Settings%5CChuanwang%20CAO%5C%E6%A1%8C%E9%9D%A2%5C%E6%91%87%E8%9A%8A%E8%BD%AC%E5%BD%95%E6%9C%AC%E6%95%B0%E6%8D%AE%E7%BB%93%E6%9E%9C%E6%95%B4%E7%90%860225%5CCK-vs-PT.upregulation%20invovled%20in%20pathway.xls#RANGE!gene211%23RANGE!gene211) | 12（0.24%） | 4（0.13%） | 0.980809 | 1.00E+00 | ko04330 |
| 212 | [Glyoxylate and dicarboxylate metabolism](../../../../C:%5CDocuments%20and%20Settings%5CChuanwang%20CAO%5C%E6%A1%8C%E9%9D%A2%5C%E6%91%87%E8%9A%8A%E8%BD%AC%E5%BD%95%E6%9C%AC%E6%95%B0%E6%8D%AE%E7%BB%93%E6%9E%9C%E6%95%B4%E7%90%860225%5CCK-vs-PT.upregulation%20invovled%20in%20pathway.xls#RANGE!gene212%23RANGE!gene212) | 0（0.00%） | 2（0.07%） | 0.983322 | 1.00E+00 | ko00630 |
| 213 | [Spliceosome](../../../../C:%5CDocuments%20and%20Settings%5CChuanwang%20CAO%5C%E6%A1%8C%E9%9D%A2%5C%E6%91%87%E8%9A%8A%E8%BD%AC%E5%BD%95%E6%9C%AC%E6%95%B0%E6%8D%AE%E7%BB%93%E6%9E%9C%E6%95%B4%E7%90%860225%5CCK-vs-PT.upregulation%20invovled%20in%20pathway.xls#RANGE!gene213%23RANGE!gene213) | 42（0.82%） | 9（0.29%） | 0.98379 | 1.00E+00 | ko03040 |
| 214 | [Non-small cell lung cancer](../../../../C:%5CDocuments%20and%20Settings%5CChuanwang%20CAO%5C%E6%A1%8C%E9%9D%A2%5C%E6%91%87%E8%9A%8A%E8%BD%AC%E5%BD%95%E6%9C%AC%E6%95%B0%E6%8D%AE%E7%BB%93%E6%9E%9C%E6%95%B4%E7%90%860225%5CCK-vs-PT.upregulation%20invovled%20in%20pathway.xls#RANGE!gene214%23RANGE!gene214) | 4（0.08%） | 1（0.03%） | 0.98518 | 1.00E+00 | ko05223 |
| 215 | [Vasopressin-regulated water reabsorption](../../../../C:%5CDocuments%20and%20Settings%5CChuanwang%20CAO%5C%E6%A1%8C%E9%9D%A2%5C%E6%91%87%E8%9A%8A%E8%BD%AC%E5%BD%95%E6%9C%AC%E6%95%B0%E6%8D%AE%E7%BB%93%E6%9E%9C%E6%95%B4%E7%90%860225%5CCK-vs-PT.upregulation%20invovled%20in%20pathway.xls#RANGE!gene215%23RANGE!gene215) | 13（0.26%） | 0（0.00%） | 0.986756 | 1.00E+00 | ko04962 |
| 216 | [Inositol phosphate metabolism](../../../../C:%5CDocuments%20and%20Settings%5CChuanwang%20CAO%5C%E6%A1%8C%E9%9D%A2%5C%E6%91%87%E8%9A%8A%E8%BD%AC%E5%BD%95%E6%9C%AC%E6%95%B0%E6%8D%AE%E7%BB%93%E6%9E%9C%E6%95%B4%E7%90%860225%5CCK-vs-PT.upregulation%20invovled%20in%20pathway.xls#RANGE!gene216%23RANGE!gene216) | 10（0.20%） | 6（0.20%） | 0.987019 | 1.00E+00 | ko00562 |
| 217 | [N-Glycan biosynthesis](../../../../C:%5CDocuments%20and%20Settings%5CChuanwang%20CAO%5C%E6%A1%8C%E9%9D%A2%5C%E6%91%87%E8%9A%8A%E8%BD%AC%E5%BD%95%E6%9C%AC%E6%95%B0%E6%8D%AE%E7%BB%93%E6%9E%9C%E6%95%B4%E7%90%860225%5CCK-vs-PT.upregulation%20invovled%20in%20pathway.xls#RANGE!gene217%23RANGE!gene217) | 7（0.14%） | 0（0.00%） | 0.98903 | 1.00E+00 | ko00510 |
| 218 | [Huntington's disease](../../../../C:%5CDocuments%20and%20Settings%5CChuanwang%20CAO%5C%E6%A1%8C%E9%9D%A2%5C%E6%91%87%E8%9A%8A%E8%BD%AC%E5%BD%95%E6%9C%AC%E6%95%B0%E6%8D%AE%E7%BB%93%E6%9E%9C%E6%95%B4%E7%90%860225%5CCK-vs-PT.upregulation%20invovled%20in%20pathway.xls#RANGE!gene218%23RANGE!gene218) | 47（0.92%） | 17（0.56%） | 0.989594 | 1.00E+00 | ko05016 |
| 219 | [Protein export](../../../../C:%5CDocuments%20and%20Settings%5CChuanwang%20CAO%5C%E6%A1%8C%E9%9D%A2%5C%E6%91%87%E8%9A%8A%E8%BD%AC%E5%BD%95%E6%9C%AC%E6%95%B0%E6%8D%AE%E7%BB%93%E6%9E%9C%E6%95%B4%E7%90%860225%5CCK-vs-PT.upregulation%20invovled%20in%20pathway.xls#RANGE!gene219%23RANGE!gene219) | 1（0.02%） | 1（0.03%） | 0.990386 | 1.00E+00 | ko03060 |
| 220 | [Mismatch repair](../../../../C:%5CDocuments%20and%20Settings%5CChuanwang%20CAO%5C%E6%A1%8C%E9%9D%A2%5C%E6%91%87%E8%9A%8A%E8%BD%AC%E5%BD%95%E6%9C%AC%E6%95%B0%E6%8D%AE%E7%BB%93%E6%9E%9C%E6%95%B4%E7%90%860225%5CCK-vs-PT.upregulation%20invovled%20in%20pathway.xls#RANGE!gene220%23RANGE!gene220) | 4（0.08%） | 1（0.03%） | 0.990418 | 1.00E+00 | ko03430 |
| 221 | [Basal transcription factors](../../../../C:%5CDocuments%20and%20Settings%5CChuanwang%20CAO%5C%E6%A1%8C%E9%9D%A2%5C%E6%91%87%E8%9A%8A%E8%BD%AC%E5%BD%95%E6%9C%AC%E6%95%B0%E6%8D%AE%E7%BB%93%E6%9E%9C%E6%95%B4%E7%90%860225%5CCK-vs-PT.upregulation%20invovled%20in%20pathway.xls#RANGE!gene221%23RANGE!gene221) | 9（0.18%） | 2（0.07%） | 0.991109 | 1.00E+00 | ko03022 |
| 222 | [Nucleotide excision repair](../../../../C:%5CDocuments%20and%20Settings%5CChuanwang%20CAO%5C%E6%A1%8C%E9%9D%A2%5C%E6%91%87%E8%9A%8A%E8%BD%AC%E5%BD%95%E6%9C%AC%E6%95%B0%E6%8D%AE%E7%BB%93%E6%9E%9C%E6%95%B4%E7%90%860225%5CCK-vs-PT.upregulation%20invovled%20in%20pathway.xls#RANGE!gene222%23RANGE!gene222) | 7（0.14%） | 3（0.10%） | 0.99207 | 1.00E+00 | ko03420 |
| 223 | [Long-term potentiation](../../../../C:%5CDocuments%20and%20Settings%5CChuanwang%20CAO%5C%E6%A1%8C%E9%9D%A2%5C%E6%91%87%E8%9A%8A%E8%BD%AC%E5%BD%95%E6%9C%AC%E6%95%B0%E6%8D%AE%E7%BB%93%E6%9E%9C%E6%95%B4%E7%90%860225%5CCK-vs-PT.upregulation%20invovled%20in%20pathway.xls#RANGE!gene223%23RANGE!gene223) | 16（0.31%） | 3（0.10%） | 0.992374 | 1.00E+00 | ko04720 |
| 224 | [Tight junction](../../../../C:%5CDocuments%20and%20Settings%5CChuanwang%20CAO%5C%E6%A1%8C%E9%9D%A2%5C%E6%91%87%E8%9A%8A%E8%BD%AC%E5%BD%95%E6%9C%AC%E6%95%B0%E6%8D%AE%E7%BB%93%E6%9E%9C%E6%95%B4%E7%90%860225%5CCK-vs-PT.upregulation%20invovled%20in%20pathway.xls#RANGE!gene224%23RANGE!gene224) | 40（0.79%） | 13（0.43%） | 0.992568 | 1.00E+00 | ko04530 |
| 225 | [Ribosome biogenesis in eukaryotes](../../../../C:%5CDocuments%20and%20Settings%5CChuanwang%20CAO%5C%E6%A1%8C%E9%9D%A2%5C%E6%91%87%E8%9A%8A%E8%BD%AC%E5%BD%95%E6%9C%AC%E6%95%B0%E6%8D%AE%E7%BB%93%E6%9E%9C%E6%95%B4%E7%90%860225%5CCK-vs-PT.upregulation%20invovled%20in%20pathway.xls#RANGE!gene225%23RANGE!gene225) | 30（0.59%） | 8（0.26%） | 0.993085 | 1.00E+00 | ko03008 |
| 226 | [Oocyte meiosis](../../../../C:%5CDocuments%20and%20Settings%5CChuanwang%20CAO%5C%E6%A1%8C%E9%9D%A2%5C%E6%91%87%E8%9A%8A%E8%BD%AC%E5%BD%95%E6%9C%AC%E6%95%B0%E6%8D%AE%E7%BB%93%E6%9E%9C%E6%95%B4%E7%90%860225%5CCK-vs-PT.upregulation%20invovled%20in%20pathway.xls#RANGE!gene226%23RANGE!gene226) | 20（0.39%） | 6（0.20%） | 0.993616 | 1.00E+00 | ko04114 |
| 227 | [Parkinson's disease](../../../../C:%5CDocuments%20and%20Settings%5CChuanwang%20CAO%5C%E6%A1%8C%E9%9D%A2%5C%E6%91%87%E8%9A%8A%E8%BD%AC%E5%BD%95%E6%9C%AC%E6%95%B0%E6%8D%AE%E7%BB%93%E6%9E%9C%E6%95%B4%E7%90%860225%5CCK-vs-PT.upregulation%20invovled%20in%20pathway.xls#RANGE!gene227%23RANGE!gene227) | 16（0.31%） | 5（0.16%） | 0.995281 | 1.00E+00 | ko05012 |
| 228 | [African trypanosomiasis](../../../../C:%5CDocuments%20and%20Settings%5CChuanwang%20CAO%5C%E6%A1%8C%E9%9D%A2%5C%E6%91%87%E8%9A%8A%E8%BD%AC%E5%BD%95%E6%9C%AC%E6%95%B0%E6%8D%AE%E7%BB%93%E6%9E%9C%E6%95%B4%E7%90%860225%5CCK-vs-PT.upregulation%20invovled%20in%20pathway.xls#RANGE!gene228%23RANGE!gene228) | 0（0.00%） | 1（0.03%） | 0.995989 | 1.00E+00 | ko05143 |
| 229 | [Chronic myeloid leukemia](../../../../C:%5CDocuments%20and%20Settings%5CChuanwang%20CAO%5C%E6%A1%8C%E9%9D%A2%5C%E6%91%87%E8%9A%8A%E8%BD%AC%E5%BD%95%E6%9C%AC%E6%95%B0%E6%8D%AE%E7%BB%93%E6%9E%9C%E6%95%B4%E7%90%860225%5CCK-vs-PT.upregulation%20invovled%20in%20pathway.xls#RANGE!gene229%23RANGE!gene229) | 10（0.20%） | 1（0.03%） | 0.996587 | 1.00E+00 | ko05220 |
| 230 | [Cell cycle](../../../../C:%5CDocuments%20and%20Settings%5CChuanwang%20CAO%5C%E6%A1%8C%E9%9D%A2%5C%E6%91%87%E8%9A%8A%E8%BD%AC%E5%BD%95%E6%9C%AC%E6%95%B0%E6%8D%AE%E7%BB%93%E6%9E%9C%E6%95%B4%E7%90%860225%5CCK-vs-PT.upregulation%20invovled%20in%20pathway.xls#RANGE!gene230%23RANGE!gene230) | 26（0.51%） | 8（0.26%） | 0.997134 | 1.00E+00 | ko04110 |
| 231 | [Glycosylphosphatidylinositol](../../../../C:%5CDocuments%20and%20Settings%5CChuanwang%20CAO%5C%E6%A1%8C%E9%9D%A2%5C%E6%91%87%E8%9A%8A%E8%BD%AC%E5%BD%95%E6%9C%AC%E6%95%B0%E6%8D%AE%E7%BB%93%E6%9E%9C%E6%95%B4%E7%90%860225%5CCK-vs-PT.upregulation%20invovled%20in%20pathway.xls#RANGE!gene231%23RANGE!gene231)（GPI）-anchor biosynthesis | 1（0.02%） | 2（0.07%） | 0.997172 | 1.00E+00 | ko00563 |
| 232 | [Homologous recombination](../../../../C:%5CDocuments%20and%20Settings%5CChuanwang%20CAO%5C%E6%A1%8C%E9%9D%A2%5C%E6%91%87%E8%9A%8A%E8%BD%AC%E5%BD%95%E6%9C%AC%E6%95%B0%E6%8D%AE%E7%BB%93%E6%9E%9C%E6%95%B4%E7%90%860225%5CCK-vs-PT.upregulation%20invovled%20in%20pathway.xls#RANGE!gene232%23RANGE!gene232) | 3（0.06%） | 2（0.07%） | 0.998466 | 1.00E+00 | ko03440 |
| 233 | [Phosphatidylinositol signaling system](../../../../C:%5CDocuments%20and%20Settings%5CChuanwang%20CAO%5C%E6%A1%8C%E9%9D%A2%5C%E6%91%87%E8%9A%8A%E8%BD%AC%E5%BD%95%E6%9C%AC%E6%95%B0%E6%8D%AE%E7%BB%93%E6%9E%9C%E6%95%B4%E7%90%860225%5CCK-vs-PT.upregulation%20invovled%20in%20pathway.xls#RANGE!gene233%23RANGE!gene233) | 14（0.27%） | 6（0.20%） | 0.998959 | 1.00E+00 | ko04070 |
| 234 | [RNA transport](../../../../C:%5CDocuments%20and%20Settings%5CChuanwang%20CAO%5C%E6%A1%8C%E9%9D%A2%5C%E6%91%87%E8%9A%8A%E8%BD%AC%E5%BD%95%E6%9C%AC%E6%95%B0%E6%8D%AE%E7%BB%93%E6%9E%9C%E6%95%B4%E7%90%860225%5CCK-vs-PT.upregulation%20invovled%20in%20pathway.xls#RANGE!gene234%23RANGE!gene234) | 50（0.98%） | 14（0.46%） | 0.999221 | 1.00E+00 | ko03013 |
| 235 | [DNA replication](../../../../C:%5CDocuments%20and%20Settings%5CChuanwang%20CAO%5C%E6%A1%8C%E9%9D%A2%5C%E6%91%87%E8%9A%8A%E8%BD%AC%E5%BD%95%E6%9C%AC%E6%95%B0%E6%8D%AE%E7%BB%93%E6%9E%9C%E6%95%B4%E7%90%860225%5CCK-vs-PT.upregulation%20invovled%20in%20pathway.xls#RANGE!gene235%23RANGE!gene235) | 6（0.12%） | 2（0.07%） | 0.999253 | 1.00E+00 | ko03030 |
| 236 | [Ubiquitin mediated proteolysis](../../../../C:%5CDocuments%20and%20Settings%5CChuanwang%20CAO%5C%E6%A1%8C%E9%9D%A2%5C%E6%91%87%E8%9A%8A%E8%BD%AC%E5%BD%95%E6%9C%AC%E6%95%B0%E6%8D%AE%E7%BB%93%E6%9E%9C%E6%95%B4%E7%90%860225%5CCK-vs-PT.upregulation%20invovled%20in%20pathway.xls#RANGE!gene236%23RANGE!gene236) | 26（0.51%） | 8（0.26%） | 0.999475 | 1.00E+00 | ko04120 |
| 237 | [Oxidative phosphorylation](../../../../C:%5CDocuments%20and%20Settings%5CChuanwang%20CAO%5C%E6%A1%8C%E9%9D%A2%5C%E6%91%87%E8%9A%8A%E8%BD%AC%E5%BD%95%E6%9C%AC%E6%95%B0%E6%8D%AE%E7%BB%93%E6%9E%9C%E6%95%B4%E7%90%860225%5CCK-vs-PT.upregulation%20invovled%20in%20pathway.xls#RANGE!gene237%23RANGE!gene237) | 8（0.16%） | 6（0.20%） | 0.999858 | 1.00E+00 | ko00190 |
| 238 | Ribosome | 4（0.08%） | 17（0.56%） | 1 | 1.00E+00 | ko03010 |
